# WTAP Transcriptional Suppression by KLF9 Drives Osteoclastogenesis via M^6^A‐Mediated Regulation of CSF1R Signaling in Estrogen‐Deficient Osteoporosis

**DOI:** 10.1002/advs.77227

**Published:** 2026-08-30

**Authors:** Chen Shen, Xin Liu, Gaoran Ge, Li Xu, Ziyu Zhang, Chang Lv, Hao Xu, Wenhao Li, Yi Qin, Qifeng Sheng, Qihan Wang, Hongxia Li, Jun Zhou, Huilin Yang, Dechun Geng

**Affiliations:** ^1^ Department of Orthopedics The First Affiliated Hospital of Soochow University Suzhou Jiangsu China; ^2^ Department of Cardiology The First Affiliated Hospital of Soochow University Suzhou Jiangsu China

**Keywords:** KLF9, m^6^A methylation, osteoclastogenesis, osteoporosis, WTAP

## Abstract

Osteoporosis, characterized by imbalanced bone homeostasis, is driven by excessive osteoclast‐mediated bone resorption, yet the epitranscriptomic regulation via m^6^A modification remains unclear. Here, we identify WTAP, a component of the m^6^A methyltransferase complex, as a critical negative regulator of osteoclastogenesis. Myeloid‐specific Wtap knockout in mice exacerbates osteoclast formation and osteoporotic bone loss. Mechanistically, WTAP mediates m^6^A deposition on Csflr mRNA, promoting degradation via the key m^6^A reader YTHDF2 and downregulating CSF1R expression, thereby enhancing osteoclastogenesis and bone loss in estrogen‐deficient osteoporosis. We further discover that KLF9, induced during osteoclast differentiation, translocates to the nucleus to directly repress Wtap transcription, initiating this pathological process. Concurrent conditional knockout of KLF9 in osteoclast precursors rescues the exacerbated osteoporotic bone loss driven by myeloid‐specific Wtap deficiency in vivo. This KLF9/WTAP/YTHDF2/m^6^A/CSF1R axis establishes a novel epigenetic circuit regulating bone resorption. Therapeutically, targeting this axis via AAV‐mediated Wtap overexpression or pharmacological CSF1R inhibition with pexidartinib effectively ameliorates bone loss in osteoporotic mice. Our findings elucidate a previously unrecognized epitranscriptomic mechanism controlling osteoclastogenesis and highlight its therapeutic potential for pathological bone resorption.

## Graphical abstract

Scheme of the KLF9/WTAP/YTHDF2/m^6^A/CSF1R regulatory axis in osteoclastogenesis and estrogen‐deficient osteoporosis. WTAP‐mediated m^6^A modification of Csf1r mRNA governs osteoclastogenesis via a YTHDF2‐mediated pathway. Pathological upregulation of KLF9 drives Wtap transcription, leading to increased m^6^A deposition on the 3’‐UTR of Csf1r mRNA. This reduces YTHDF2 binding affinity, destabilizing Csf1r mRNA and elevating CSF1R protein levels upon M‐CSF stimulation, thereby enhancing osteoclastogenesis and promoting bone resorption. Conversely, therapeutic inhibition of KLF9 or WTAP decreases m^6^A modification on CSF1R, restores YTHDF2 binding, stabilizes Csf1r mRNA, and reduces CSF1R expression, ultimately suppressing osteoclast differentiation. Pharmacological blockade of CSF1R with PLX3397 further attenuates osteoclastogenesis, providing a strategy to alleviate estrogen‐deficient osteoporosis.

## 1 Introduction

Osteoporosis (OP) is a major global public health problem, affecting hundreds of millions of individuals worldwide, with postmenopausal women and the elderly being disproportionately at risk [1]. Fragility fractures resulting from the disease significantly increase morbidity, mortality, and impose substantial economic costs for human society [2]. The pathological characterization of OP lies in the imbalance in bone remodeling, where excessive bone resorption driven by osteoclasts plays a decisive role [3]. Osteoclasts, multinucleated giant cells derived from monocyte/macrophage precursors, are abnormally activated, and their differentiation is precisely regulated by the M‐CSF/CSF1R signaling, RANKL/RANK/OPG axis, and a network of local factors and cellular interactions [4, 5]. The dysregulated coupling between osteoblasts and osteoclasts leads to bone resorption outpacing formation, resulting in progressive bone loss and microarchitectural deterioration [6]. Therefore, a deep understanding of osteoclast differentiation, maturation, and its regulatory network is essential for elucidating the pathogenesis of OP and developing novel clinical therapies [7].

N6‐methyladenosine (m^6^A), the most prevalent internal RNA modification in eukaryotes, plays a vital role in post‐transcriptional gene regulation and is increasingly recognized as a key modulator of bone homeostasis [8]. The dynamic and reversible m^6^A modification, installed by methyltransferase complexes (Writers), removed by demethylases (Erasers), and interpreted by binding proteins (Readers), regulates mRNA fate, including stability, splicing, export, and translation, thereby determining the differentiation and function of both osteoblasts and osteoclasts [9, 10, 11]. Among them, the METTL3‐METTL14‐WTAP complex constitutes the core m^6^A “Writer” complex and plays a significant role in regulating osteoclast differentiation [12]. However, the existing literature presents seemingly conflicting conclusions regarding the specific functions of its individual components in this process [13, 14].

Wilms’ tumor 1‐associating protein (WTAP) serves as a critical scaffold component of the m^6^A methyltransferase complex, essential for its nuclear localization and catalytic activity [15]. In the skeletal system, WTAP‐mediated m^6^A deposition has been implicated in regulating key transcripts involved in bone metabolism [16]. In osteoblastic lineage cells, WTAP is involved in regulating genes critical for bone formation [17]. However, the role of WTAP in osteoclast differentiation and OP is unclear. In addition, while extensive research has focused on how m^6^A modifications regulate downstream target genes, the upstream mechanisms that precisely control the expression of the m^6^A regulatory components themselves, especially their transcriptional regulation, remain largely unexplored.

Here, we studied the function and underlying mechanism of WTAP in osteoclast differentiation and OP progression. Through integrated multi‐omics analysis, cellular and molecular biology, and genetic mouse models, we identified and validated a previously unrecognized KLF9/WTAP/YTHDF2/m^6^A/CSF1R regulatory axis. Specifically, we demonstrate that the transcription factor KLF9 is upregulated and undergoes nuclear translocation during osteoclastogenesis, where it directly binds to and represses WTAP transcription. The consequent downregulation of WTAP reduces m^6^A deposition on the mRNA of the critical osteoclastogenic factor Csf1r, which attenuates its degradation mediated by the m^6^A reader YTHDF2. This post‐transcriptional stabilization of Csf1r mRNA drives excessive osteoclast formation and bone loss. Our study identifies a new pathogenic signaling pathway in OP, from upstream transcriptional repression of a core m^6^A writer to the downstream stabilization of a key osteoclast receptor. It offers a novel mechanistic approach for therapeutic strategies aimed at the m^6^A epitranscriptome in bone disease.

## 2 Results

### 2.1 WTAP Expression Was Decreased During Osteoclastogenesis in OP Patients and OVX Mice

To investigate the role of WTAP in the pathogenesis of osteoporosis (OP), we performed an integrated analysis of single‐cell RNA sequencing data (GSE147287) derived from OP patient samples (Figure 1A). WTAP expression was predominantly enriched in discrete subpopulations of bone marrow mesenchymal stem cells (BMSCs) and macrophages, with additional lower‐level expression observed in neutrophils, myelocytes, and other immune cell subsets (Figure 1B). This distribution closely paralleled the expression patterns of osteoclast markers, including Ctsk and Nfatc1, suggesting a potential involvement of WTAP in osteoclast‐driven bone resorption in OP. Pseudotemporal trajectory analysis further revealed that, in contrast to the progressive upregulation of Ctsk and Nfatc1 during osteoclast differentiation, WTAP expression gradually declined in osteoclast precursor cells (Figure 1C). Consistent with established roles as m^6^A methyltransferase (“Writers”) components, Mettl3 and Mettl14 exhibited subpopulation‐specific localization patterns in line with previous studies (Figure S1A) [18, 19]. Notably, their expression profiles diverged within macrophage lineages: Mettl3 displayed an increasing trend, whereas Mettl14 expression showed a concomitant decrease (Figure S1B). Moreover, Wtap expression exhibited an increase during BMSCs differentiation, implying a potential functional role in osteoblast‐driven bone formation, which aligned with prior research (Figure S1C) [20].

**FIGURE 1:**
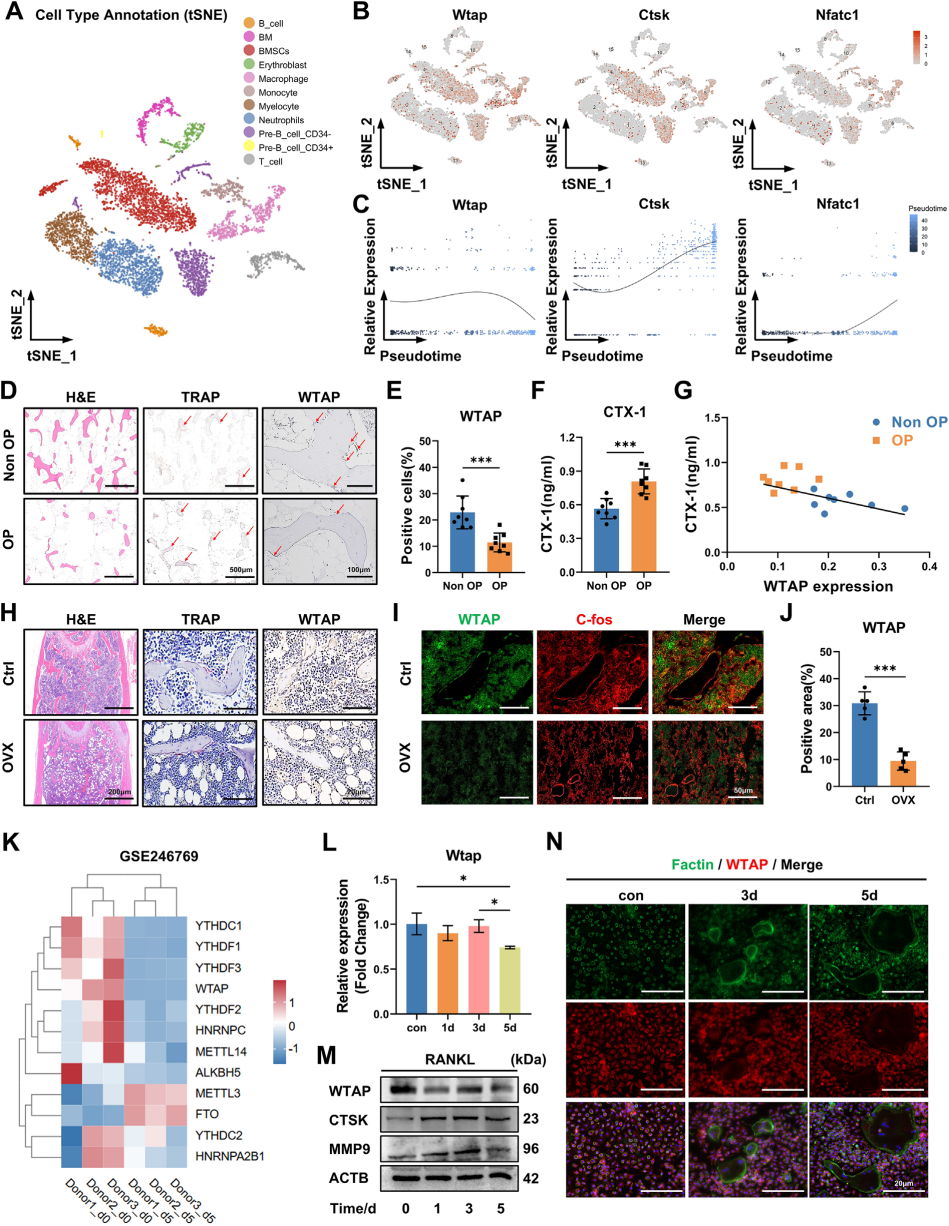
WTAP expression was decreased during osteoclastogenesis in OP patients and OVX mice. (A) tSNE plot of single‐cell RNA sequencing data (GSE147287) from OP patient bone marrow, annotated for major cell types. (B) Expression levels of WTAP, Ctsk, and Nfatc1 mapped onto the tSNE plot. (C) Pseudotemporal trajectory analysis showing the relative expression of WTAP, Ctsk, and Nfatc1 during osteoclast differentiation. (D) Representative H&E, TRAP, and WTAP immunohistochemical staining images of bone tissue sections from non‐OP and OP patients. Scale bars: 500 µm (H&E, TRAP) and 100 µm (WTAP). (E) Quantification of WTAP‐positive cells in bone tissue sections from non‐OP and OP patients (*n* = 8 per group). (F) Serum CTX‐1 levels in non‐OP and OP patients (*n* = 8 per group). (G) Correlation analysis between WTAP expression in bone tissue and serum CTX‐1 levels. (H) Representative H&E, TRAP, and WTAP immunohistochemical staining images of bone tissue sections from sham‐operated (Ctrl) and OVX mice. Scale bars: 200 µm (H&E, TRAP) and 50 µm (WTAP). (I) Representative immunofluorescence images showing co‐localization of WTAP (green) and C‐fos (red) in bone tissue sections from Ctrl and OVX mice. Scale bars, 50 µm. (J) Quantification of WTAP‐positive area in bone tissue sections from Ctrl and OVX mice (*n* = 5 per group). (K) Heatmap showing the expression of m^6^A‐related genes during RANKL‐induced osteoclast differentiation (GSE246769). (L) qRT‐PCR analysis of Wtap expression in BMDMs at different time points after RANKL induction. (M) Western blot of WTAP, CTSK, MMP9, and ACTB protein levels in BMDMs at different time points after RANKL induction. (N) Representative immunofluorescence images showing WTAP (red) expression in BMDMs at different time points after RANKL stimulation. F‐actin was stained with phalloidin (green). Scale bars, 20 µm. Data are presented as mean ± SEM. Statistical significance was determined by unpaired two‐tailed Student's *t*‐test (E, F, J) or one‐way ANOVA with Tukey's post hoc test (L). ^*^ *p* < 0.05, ^***^ *p* < 0.001.

Bone tissue samples were obtained from sixteen patients undergoing total knee arthroplasty, stratified into OP and non‐OP groups based on dual‐energy X‐ray absorptiometry (DEXA)‐determined bone mineral density (BMD). Immunohistochemical staining revealed a marked reduction in WTAP‐positive cells within bone tissue sections from OP patients (Figure 1D,E) with a higher level of serum C‐terminal telopeptide of type I collagen (CTX‐1) (Figure 1F). Correlation analysis revealed an inverse relationship between WTAP immunostaining intensity in bone tissue and serum CTX‐1 levels (Figure 1G). These findings collectively indicated that reduced WTAP expression in bone tissue was closely correlated with elevated bone resorption markers in osteoporotic patients, consistent with a potential role for WTAP in osteoclast‐mediated bone loss.

To further investigate the role of WTAP in vivo, we established an ovariectomy (OVX)‐induced osteoporosis mouse model. A significant reduction was confirmed in WTAP‐positive signals within the bone tissue of OVX mice compared to sham‐operated controls (Figure 1H; Figure S1D). Consistent with the human data, WTAP expression levels in mouse bone tissue demonstrated a negative correlation with circulating CTX‐1 concentrations (Figure S1E,F). Furthermore, co‐localization studies in mouse bone tissue revealed that WTAP signals were predominantly present in C‐fos‐positive cells (Figure 1I), which suggested that the distribution and potentially the function of WTAP in these osteoclast precursor populations diminished as osteoporosis progressed (Figure 1J).

We then analyzed bulk RNA sequencing data (GSE246769) from RANKL‐induced osteoclast differentiation and found a significant downregulation of WTAP expression post‐induction (Figure 1K). This decrease in WTAP was subsequently validated at both the mRNA and protein levels in primary BMDMs cultures (Figure 1L,M; Figure S2A,C,D). Immunofluorescence also corroborated the time‐dependent downregulation of WTAP during RANKL‐induced osteoclast differentiation (Figure 1N). Consistent with our prior bioinformatic analyses, both Mettl3 and Mettl14 exhibited dynamic expression changes during osteoclastogenesis (Figure S2B). Interestingly, despite the marked changes of “Writers”, the global m^6^A modification levels in BMDMs remained relatively stable throughout RANKL‐induced osteoclast differentiation (Figure S2E,F), which suggested a dynamic equilibrium in m^6^A methylation during this cellular process.

### 2.2 Intervention of WTAP Inversely Regulated Osteoclastogenesis and OP Pathogenesis In Vitro and In Vivo

To further explore the functional role of WTAP in osteoclastogenesis, we intervened in BMDMs using siRNA‐mediated knockdown and plasmid‐mediated overexpression, respectively (Figure 2A,F). Knockdown of WTAP resulted in a significant upregulation of key osteoclastogenic genes (Nfatc1, Ctsk, Mmp9) as quantified by qPCR (Figure 2B). This transcriptional induction was confirmed at the protein level, with Western blot analysis demonstrating a similar increase in CTSK and MMP9 expression (Figure 2C; Figure S3A,B). Functionally, Wtap knockdown enhanced osteoclast formation, as evidenced by an increased percentage of multinucleated cells in TRAP staining (Figure 2D; Figure S3C), and a greater area of cell fusion in immunofluorescence assays (Figure 2E; Figure S3D). In contrast, plasmid‐driven overexpression of WTAP in BMDMs led to a downregulation of osteoclastogenic markers (Nfatc1, Ctsk, Mmp9) at both the mRNA (Figure 2G) and protein levels (Figure 2H; Figure S3E,F). This molecular suppression translated into a functional inhibition of osteoclastogenesis, as demonstrated by a significant reduction in the formation of multinucleated TRAP‐positive cells (Figure 2I; Figure S3G) and a decreased area of cellular fusion (Figure 2J; Figure S3H). We then performed bone resorption pit assays to assess whether modulated WTAP expression affected the bone‐resorptive function of mature osteoclasts. BMDMs transfected with siWtap formed larger and more numerous resorption pits compared with the Vector control, whereas WTAP‐overexpressing BMDMs showed a marked reduction in pit area (Figure S3I). Taken together, these data indicated that loss of Wtap promoted osteoclast differentiation, while gain of Wtap inhibited osteoclastogenesis, therefore influenced osteoclast‐mediated bone resorption activity. It is noteworthy that after intervention with siWtap on BMSCs, both the osteogenic differentiation and mineralization ability of BMSCs decreased (Figure S4A,B), which is consistent with scRNA‐seq analysis and previous studies [16, 17].

**FIGURE 2:**
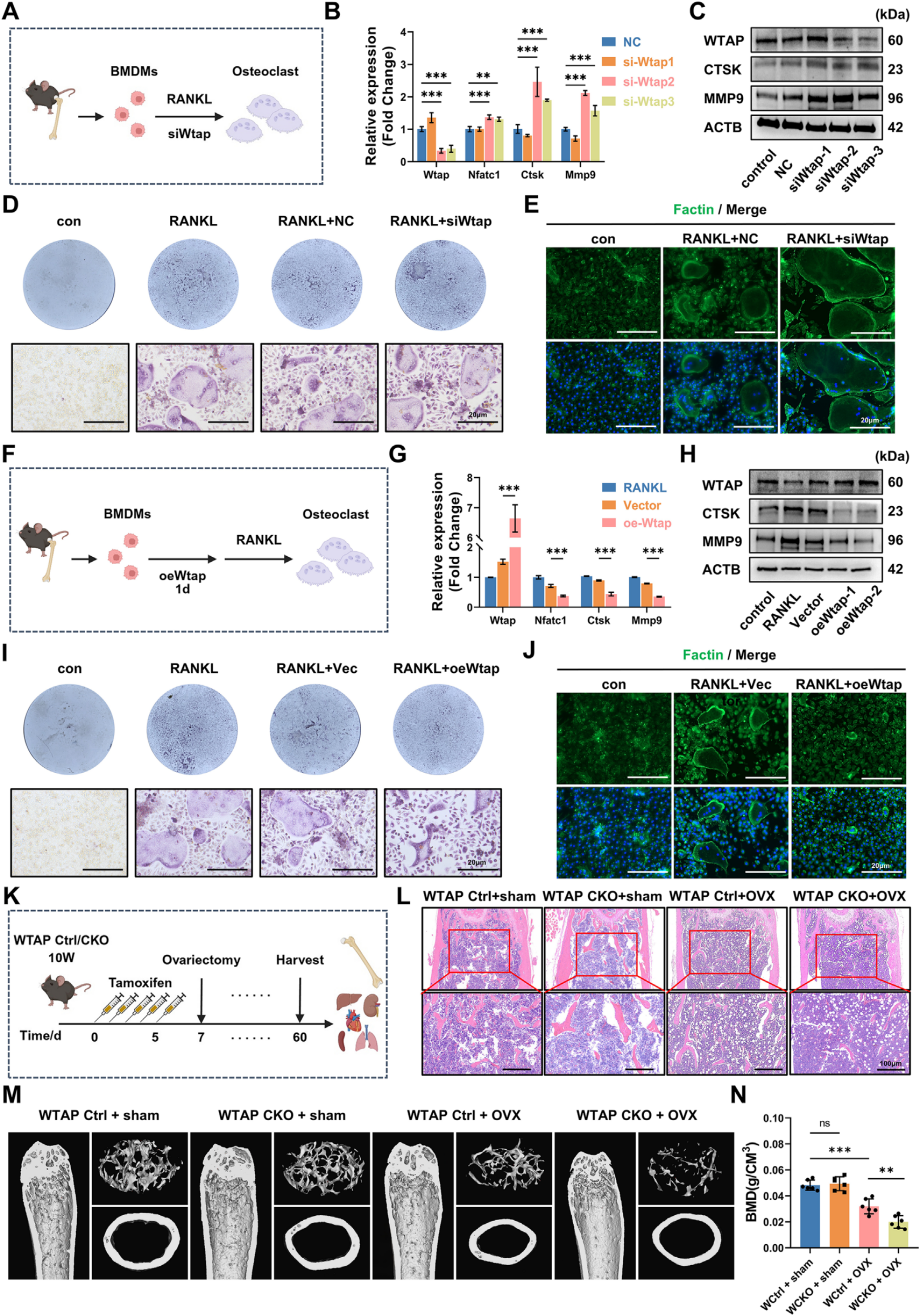
Intervention of WTAP inversely regulated osteoclastogenesis and OP pathogenesis in vitro and in vivo. (A) Schematic diagram of the experimental design for Wtap knockdown in BMDMs using siRNA. (B) qRT‐PCR analysis showing the relative expression of WTAP, Nfatc1, Ctsk, and Mmp9 in BMDMs transfected with NC or Wtap siRNAs. (C) Western blot analysis confirming the protein levels of WTAP, CTSK, and MMP9 in BMDMs after Wtap knockdown. (D) Representative TRAP staining images and corresponding microscopic views showing osteoclast formation in BMDMs under siWtap intervention. Scale bars, 20 µm. (E) Representative immunofluorescence images showing F‐actin ring formation (green) in BMDMs. Scale bars, 20 µm. (F) Schematic diagram of the experimental design for Wtap overexpression in BMDMs using plasmid transfection. (G) qRT‐PCR analysis showing the relative expression of WTAP, Nfatc1, Ctsk, and Mmp9 in BMDMs transfected with a vector control or WTAP overexpression plasmid. (H) Western blot analysis confirming the protein levels of WTAP, CTSK, and MMP9 in BMDMs after Wtap overexpression. (I) Representative TRAP staining images and corresponding microscopic views showing osteoclast formation in BMDMs under oeWtap intervention. Scale bars, 20 µm. (J) Representative immunofluorescence images showing F‐actin ring formation (green) in BMDMs. Scale bars, 20 µm. (K) Schematic diagram of the generation of myeloid lineage‐specific Wtap conditional knockout (WTAP CKO) mice and the experimental timeline for OVX and tamoxifen induction. (L) Representative H&E staining images of femur sections from WTAP Ctrl and WTAP CKO mice treated with sham or OVX surgery. Scale bars, 100 µm. (M) Representative micro‐CT images of the distal femur from WTAP Ctrl and WTAP CKO mice treated with sham or OVX surgery. (N) Quantification of bone mineral density (BMD) in the distal femur of WTAP Ctrl and WTAP CKO mice subjected to sham or OVX surgery. Data are presented as mean ± SEM. Statistical significance was determined by one‐way ANOVA with Tukey's post hoc test (B, G, N). ^*^ *p* < 0.05, ^**^ *p* < 0.01, ^***^ *p* < 0.001.

In order to further investigate the in vivo function of WTAP in osteoclastogenesis and OP progression, we generated myeloid lineage‐specific WTAP conditional knockout mice (WTAP CKO) by crossing Wtap^flox/flox^ mice with Lyz2‐CreERT2 mice (Figure S5A,B). Conditional knockout was induced by intraperitoneal injection of tamoxifen (Figure 2K), with Wtap^flox/+^; Lyz2‐CreERT2 mice serving as the control group (WTAP Ctrl). The knockout efficiency in mice was verified by Western blotting (Figure S5C) and immunofluorescence (Figure S5D) after tamoxifen induction. No visceral organ damage or abnormalities were observed during the modeling of OVX‐induced OP (Figure S5E). H&E staining of bone tissue sections revealed a more severe osteoporotic phenotype, characterized by diminished trabecular bone mass and increased marrow space in WTAP CKO mice compared to that of WTAP Ctrl mice (Figure 2L). TRAP staining and immunohistochemistry for MMP9 also revealed an increase in osteoclastogenesis and osteoclast activity (Figure S5F,G). BMDMs isolated from WTAP CKO mice exhibited an enhanced capacity for RANKL‐induced osteoclast differentiation in vitro (Figure S5H). Consistently, BMDMs isolated from WTAP CKO mice exhibited significantly enhanced resorptive activity relative to WTAP Ctrl controls (Figure S5I). In parallel, serum levels of CTX‐1 were markedly elevated in WTAP CKO mice relative to WTAP Ctrl mice (Figure S5J). Three‐point bending tests revealed that WTAP CKO mice exhibited a significant reduction in femoral maximum load compared with WTAP Ctrl mice under OVX conditions (Figure S5K), confirming that WTAP deficiency compromised bone strength. Micro‐CT of the distal femur confirmed the severe bone loss phenotype in WTAP CKO mice (Figure 2M), including lower bone mineral density (BMD), reduced trabecular bone volume fraction (BV/TV), decreased trabecular number (Tb.N), and increased trabecular separation (Tb.Sp) (Figure 2N, Figure S5L–N). No difference in cortical bone thickness (Ct.Th) was shown (Figure S5O).

We next assessed age‐related bone loss (Figure S6A). Histological analysis did not reveal a significant exacerbation of the osteoporotic phenotype in aged WTAP CKO mice of either sex compared to WTAP Ctrl mice (Figure S6B). Micro‐CT quantification (Figure S6C) indicated a non‐significant trend toward further reductions in trabecular bone parameters in aged WTAP CKO mice (Figure S6E–I). Furthermore, serum CTX‐1 levels were slightly elevated in aged WTAP CKO mice (Figure S6D). Collectively, these observations suggest that the regulatory role of WTAP in bone mass may be more pronounced in estrogen‐deficient osteoporosis than in the context of physiological, age‐related bone loss.

### 2.3 WTAP‐Dependent m^6^A Modification Targeted CSF1R to Regulate Osteoclastogenesis

To elucidate the molecular mechanism underlying WTAP‐mediated regulation of osteoclast differentiation, we performed integrated transcriptomic sequencing (RNA‐seq) and m^6^A methylomic sequencing (MeRIP‐seq) on BMDMs overexpressing Wtap (Figure 3A). Global m^6^A modification levels increased following Wtap overexpression (Figure 3B). Pie chart and metagenomic analysis revealed a predominant enrichment in 3'‐UTR (29.0%), followed by stop codon regions (28.8%) and CDS regions (27.4%) (Figure 3C,D). Differential gene expression analysis (|log_2_ (fold change)| ≥ 1, *p* < 0.05) identified 1088 significantly upregulated and 1606 significantly downregulated transcripts upon Wtap overexpression (Figure 3E). Concurrently, differential m^6^A methylation analysis (|log_2_ (fold change)| ≥ 1, *p* < 0.05) identified 1163 genes with significantly increased m^6^A peak intensity and 1152 genes with significantly decreased m^6^A peak intensity (Figure 3F). Integration of the transcriptomic and methylomic datasets identified 390 genes that exhibited significant, concordant changes in both mRNA expression and m^6^A modification levels (Figure 3G). From this integrated gene set, we screened candidates with high relevance to osteoclast differentiation and function, including CSF1R, Oscar, Il1r2, Cxcr2, F5, and Fut7 (Figure 3H). The colony‐stimulating factor 1 receptor (CSF1R), which exhibited the most pronounced changes in both datasets, emerged as a top candidate for further investigation (Figure S7A). CSF1R is a master regulator of osteoclastogenesis. It binds to its ligand CSF1 (M‐CSF), initiating a critical signaling cascade that promotes the survival, proliferation, and differentiation of osteoclast precursors into mature osteoclasts [21]. Genetic ablation or pharmacological inhibition results in osteoclast maturation obstruction and, clinically, osteopetrosis [22, 23]. We observed that WTAP overexpression led to transcriptional downregulation and m^6^A hypermethylation of Csf1r mRNA, with the methylation peak specifically enriched in its 3’UTR. This inverse correlation suggests that WTAP‐mediated m^6^A deposition promoted CSF1R mRNA decay, thereby reducing its stability and expression.

**FIGURE 3:**
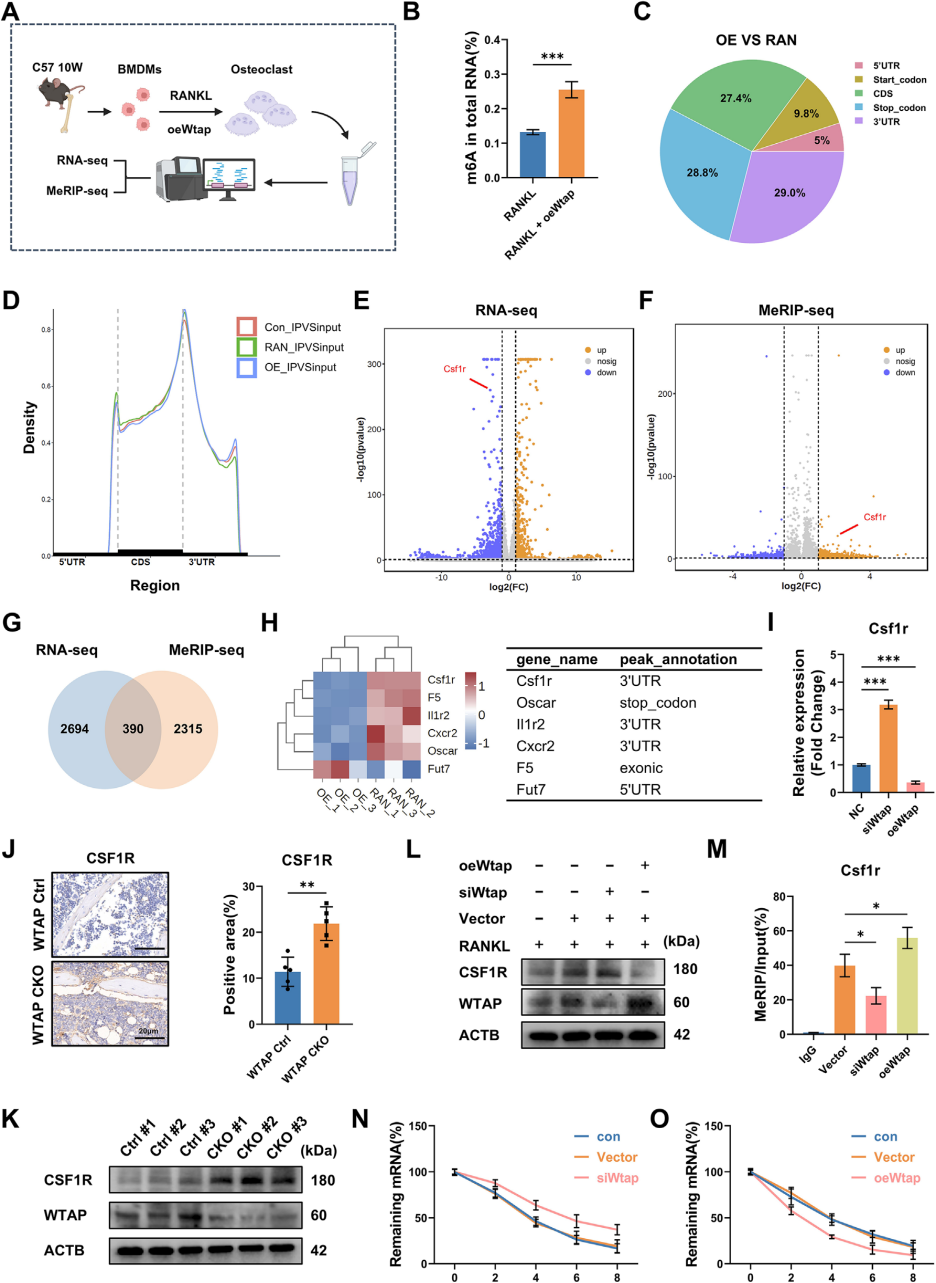
WTAP‐dependent m^6^A modification targeted Csf1r to regulate osteoclastogenesis. (A) Schematic diagram illustrating the experimental design for integrated transcriptomic (RNA‐seq) and m^6^A methylomic (MeRIP‐seq) sequencing in BMDMs overexpressing WTAP. (B) Quantification of relative m^6^A modification in total RNA from BMDMs treated with RANKL or in combination with Wtap overexpression. (C) Pie chart showing the distribution of identified m^6^A peaks across different gene regions upon Wtap overexpression. (D) Metagenetic profile showing the relative distribution of m^6^A peaks along gene transcripts. (E) Volcano plot showing the distribution of differentially expressed genes (DEGs) in BMDMs upon WTAP overexpression (|log_2_ (fold change)| ≥ 1, *p* < 0.05). The red arrow indicates Csf1r. (F) Volcano plot showing the distribution of genes with differentially methylated m^6^A peaks (DMRs) in BMDMs upon Wtap overexpression (|log_2_ (fold change)| ≥ 1, *p* < 0.05). The red arrow indicates CSF1R. G. Venn diagram showing the overlap between DEGs and genes harboring DMRs upon WTAP overexpression. (H) Heatmap showing the expression levels and m^6^A peak intensities for a set of candidate osteoclast‐related genes identified from the integrated analysis. I. qRT‐PCR analysis of CSF1R expression in BMDMs under WTAP knockdown or overexpression. (J) Representative immunohistochemical staining images of CSF1R in bone tissue sections from WTAP Ctrl and WTAP CKO mice. Scale bars, 100 µm. (K) Western blot analysis of CSF1R protein levels in BMDMs derived from WTAP Ctrl and WTAP CKO mice. (L) Quantification of CSF1R protein levels upon Wtap knockdown or overexpression. (M) MeRIP‐qPCR analysis showing the relative enrichment of Csf1r mRNA with m^6^A modification under Wtap knockdown or overexpression. (N, O) mRNA stability analysis using Actinomycin D chase assay, showing the decay kinetics of CSF1R mRNA in BMDMs following Wtap knockdown or overexpression. Data are presented as mean ± SEM. Statistical significance was determined by unpaired two‐tailed Student's *t*‐test (I, J) or one‐way ANOVA with Tukey's post hoc test (M, N, O). ^*^ *p* < 0.05, ^**^ *p* < 0.01, ^***^ *p* < 0.001.

We also performed RNA‐seq on BMDMs isolated from WTAP CKO mice (Figure S7B). Differential gene expression analysis confirmed a significant upregulation of CSF1R mRNA following Wtap ablation (Figure S7C). A heatmap of the top 50 differentially expressed genes placed CSF1R among the most prominently upregulated transcripts, a result that was consistent with and reciprocal to the downregulation observed upon Wtap overexpression (Figure S7D). Gene Ontology (GO) enrichment analysis of genes regulated upon Wtap knockout revealed enrichment for biological processes and molecular functions including cell proliferation, cytoskeleton reorganization, signaling receptor activity, and kinase activity (Figure S7E). This functional profile strongly implicated Wtap depletion in the potentiation of receptor‐mediated signaling pathways that drove osteoclast differentiation.

We then established experiments to demonstrate the regulatory role of WTAP on CSF1R. In BMDMs, siRNA‐mediated knockdown of WTAP promoted CSF1R expression, whereas plasmid‐mediated Wtap overexpression suppressed it (Figure 3I). This regulation was confirmed in vivo, where immunohistochemical staining and Western blot analysis of bone tissue revealed a significant upregulation of CSF1R protein in WTAP CKO mice compared to WTAP Ctrl mice (Figure 3J,K). At the protein level, WTAP intervention yielded results similar to those at the mRNA level, consistent with the osteoclast differentiation phenotype previously observed after WTAP intervention (Figure 3L). MeRIP‐qPCR analysis demonstrated that Wtap knockdown reduced, while Wtap overexpression increased, the m^6^A modification level on Csf1r mRNA (Figure 3M). Correspondingly, Actinomycin D chase assays revealed that Wtap depletion effectively stabilized Csf1r mRNA (Figure 3N), whereas Wtap overexpression markedly accelerated its degradation (Figure 3O).

To determine whether the osteoclastogenic effect of Wtap knockdown was mediated through CSF1R, we performed a rescue experiment in which BMDMs were co‐transfected with siWtap and siCsf1r prior to RANKL stimulation. Quantitative PCR analysis revealed that knockdown of CSF1R partially reversed the siWtap‐induced upregulation of osteoclast marker genes, including Nfatc1, Ctsk, and Mmp9 (Figure S8A). Western blot confirmed that CSF1R knockdown attenuated the elevation of CTSK and MMP9 protein levels in siWTAP‐treated cells (Figure S8B). Functionally, TRAP staining and quantification showed that co‐knockdown of CSF1R significantly rescued the enhanced osteoclast formation driven by Wtap knockdown (Figure S8C). Collectively, these data provided direct evidence that WTAP governed osteoclastogenesis through a CSF1R‐dependent pathway.

We then verified whether the regulatory role of WTAP is conserved in humans; experiments using human peripheral blood monocytes (hPBMCs) were performed. CD14^+^ monocytes from healthy donors were transfected with siWTAP or a Wtap overexpression plasmid and differentiated into osteoclasts. qRT‐PCR analysis revealed that Wtap knockdown upregulated Nfatc1, Ctsk, Mmp9, and CSF1R mRNA levels, whereas Wtap overexpression suppressed these transcripts (Figure S9A). Western blot confirmed corresponding changes at the protein level (Figure S9B). TRAP staining showed that Wtap knockdown enhanced, while Wtap overexpression impaired, human osteoclast formation (Figure S9C). These results indicated that Wtap negatively regulated human osteoclastogenesis, consistent with our murine data. All these results indicated that WTAP‐dependent m^6^A methylation targeted Csf1r transcripts for decay, and ultimately influenced osteoclast differentiation and function by affecting the maturation of osteoclast precursors.

### 2.4 WTAP Governed Osteoclastogenesis via YTHDF2‐Mediated Regulation of CSF1R mRNA Stability

The m^6^A “Readers” proteins recognize and bind to m^6^A modifications on RNA, subsequently recruiting effector complexes to execute diverse post‐transcriptional regulatory functions, including mRNA splicing, stability, translation, and decay [24]. To identify the m^6^A “Readers” protein mediating the Wtap‐dependent regulation of CSF1R, we performed an integrated analysis of previous sequencing data, focusing on the expression and correlation patterns of key “Readers” families, including YTHDC, YTHDF, IGF2BP, and HNRNP proteins (Figure 4A). Bioinformatic analysis identified YTHDF2 as the m^6^A “Reader” with the strongest statistical association and relevance to the observed regulatory network. YTH N6‐methyladenosine RNA Binding Protein F2 (YTHDF2) is a m^6^A “Reader” protein primarily known for its role in promoting the degradation of m^6^A‐modified mRNAs [25]. It often functions as a brake on osteoclast differentiation by degrading key osteoclastogenic transcripts, including Traf6, Map4k4, and Nfatc1 [26].

**FIGURE 4:**
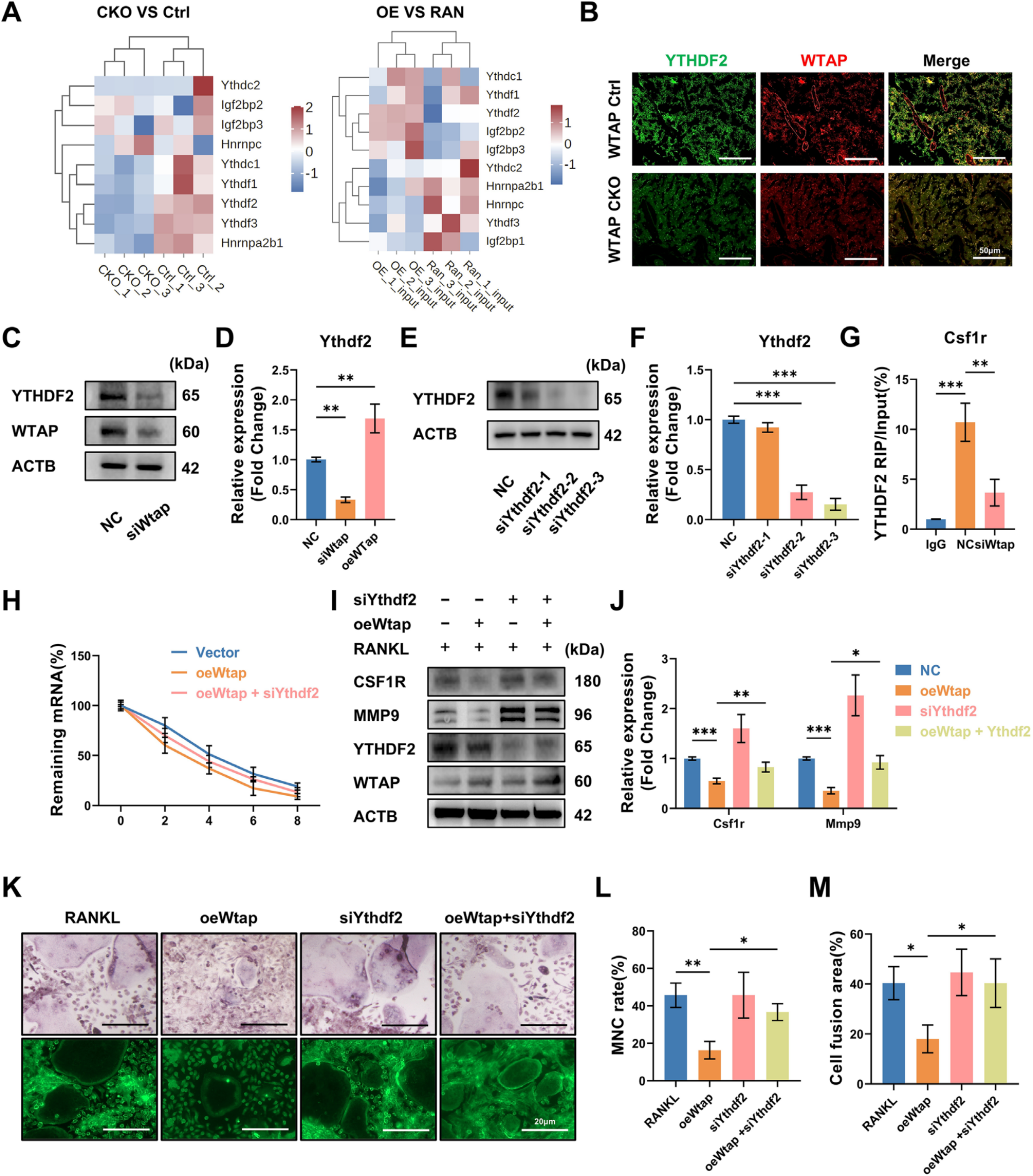
WTAP governed osteoclastogenesis via YTHDF2‐mediated regulation of Csf1r mRNA stability. (A) Heatmap showing the expression patterns of m^6^A reader proteins (YTHDC, YTHDF, IGF2BP, HNRNP families) in BMDMs from WTAP CKO mice compared to WTAP Ctrl mice and in BMDMs overexpressing Wtap compared to control. (B) Representative immunofluorescence images showing the colocalization of YTHDF2 (green) and WTAP (red) in bone tissue sections from WTAP Ctrl and WTAP CKO mice. Scale bars: 50 µm. (C) Western blot analysis showing the expression levels of YTHDF2 protein in BMDMs under Wtap knockdown. (D) qRT‐PCR analysis showing the expression levels of YTHDF2 mRNA in BMDMs under Wtap knockdown and overexpression. (E, F) Western blot and qRT‐PCR analysis validating the efficiency of Ythdf2 knockdown using siRNA in BMDMs. (G) RIP‐PCR assay showing the binding of YTHDF2 to CSF1R mRNA in BMDMs under WTAP knockdown or overexpression. (H) mRNA stability analysis using Actinomycin D chase assay, showing the decay kinetics of CSF1R mRNA in BMDMs overexpressing Wtap with or without Ythdf2 knockdown. (I, J) Western blot and qRT‐PCR analysis showing the expression levels of CSF1R, MMP9, and YTHDF2 in BMDMs under specific interventions. (K) Representative TRAP staining and immunofluorescence images of osteoclasts formed under specific interventions. Scale bars, 20 µm. (L, M) Quantification of MNC rate and cell fusion area under specific interventions. Data are presented as mean ± SEM. Statistical significance was determined by one‐way ANOVA with Tukey's post hoc test (D, F, G, J, L, M). ^*^ *p* < 0.05, ^**^ *p* < 0.01, ^***^ *p* < 0.001.

In WTAP CKO mice, the fluorescence colocalization signal of YTHDF2 and WTAP in bone tissue was significantly reduced compared to WTAP Ctrl mice (Figure 4B). In vitro, the expression of YTHDF2 protein decreased synchronously with Wtap knockdown (Figure 4C), together with mRNA levels with Wtap manipulation (Figure 4D). We then investigated whether the WTAP‐CSF1R regulatory axis is functionally dependent on YTHDF2. SiRNA was used to perform knockdown of Ythdf2 in our cellular model (Figure 4E,F). RIP‐PCR using a YTHDF2 antibody confirmed the direct binding of YTHDF2 to Csf1r mRNA. Notably, this interaction was significantly attenuated following knockdown of WTAP (Figure 4G). Actinomycin D chase assays demonstrated that the accelerated decay of CSF1R mRNA induced by WTAP overexpression was effectively rescued upon co‐knockdown of Ythdf2 (Figure 4H). This indicated that the mRNA degradation effect by WTAP mediated m^6^A modification was dependent on YTHDF2. Furthermore, at both transcript and protein levels, the downregulation of CSF1R driven by WTAP overexpression was reversed upon co‐knockdown of Ythdf2. This rescue of CSF1R expression was accompanied by a corresponding restoration in the expression of osteoclastogenic markers, such as MMP9 (Figure 4I,J). Correspondingly, functional assays confirmed this reversal: TRAP staining and immunofluorescence analysis showed that the suppression of multinucleated osteoclast formation and cell fusion caused by Wtap overexpression was largely rescued when Ythdf2 was knocked down at the same time (Figure 4K–M). To sum up, these data suggested that WTAP regulated CSF1R expression and osteoclast differentiation primarily via a YTHDF2‐mediated pathway. The core mechanism involves WTAP‐mediated m^6^A deposition on Csflr mRNA, which is subsequently recognized by the key m^6^A reader YTHDF2 to promote transcript degradation.

### 2.5 KLF9 May Act Upstream of WTAP to Regulate Osteoclastogenesis and OP Progression

Given the transcriptional changes of WTAP observed during OP progression and RANKL‐induced osteoclastogenesis, we hypothesized that its expression was controlled by upstream transcriptional regulators. We conducted an integrated bioinformatic analysis by intersecting RNA‐seq data from osteoclast differentiation, scRNA‐seq data from osteoclast precursor cells, and transcription factor binding site predictions from the UCSC genome database. This multi‐layered screen identified the transcription factor KLF9 as a potential upstream regulator of Wtap (Figure 5A). KLF9 is a member of the Krüppel‐like factor (KLF) family of transcription factors implicated in diverse processes including cell proliferation, differentiation, and metabolism [27]. While KLF9 has been studied in other cellular contexts, its specific role and regulatory targets in the program of osteoclast differentiation are largely undefined.

**FIGURE 5:**
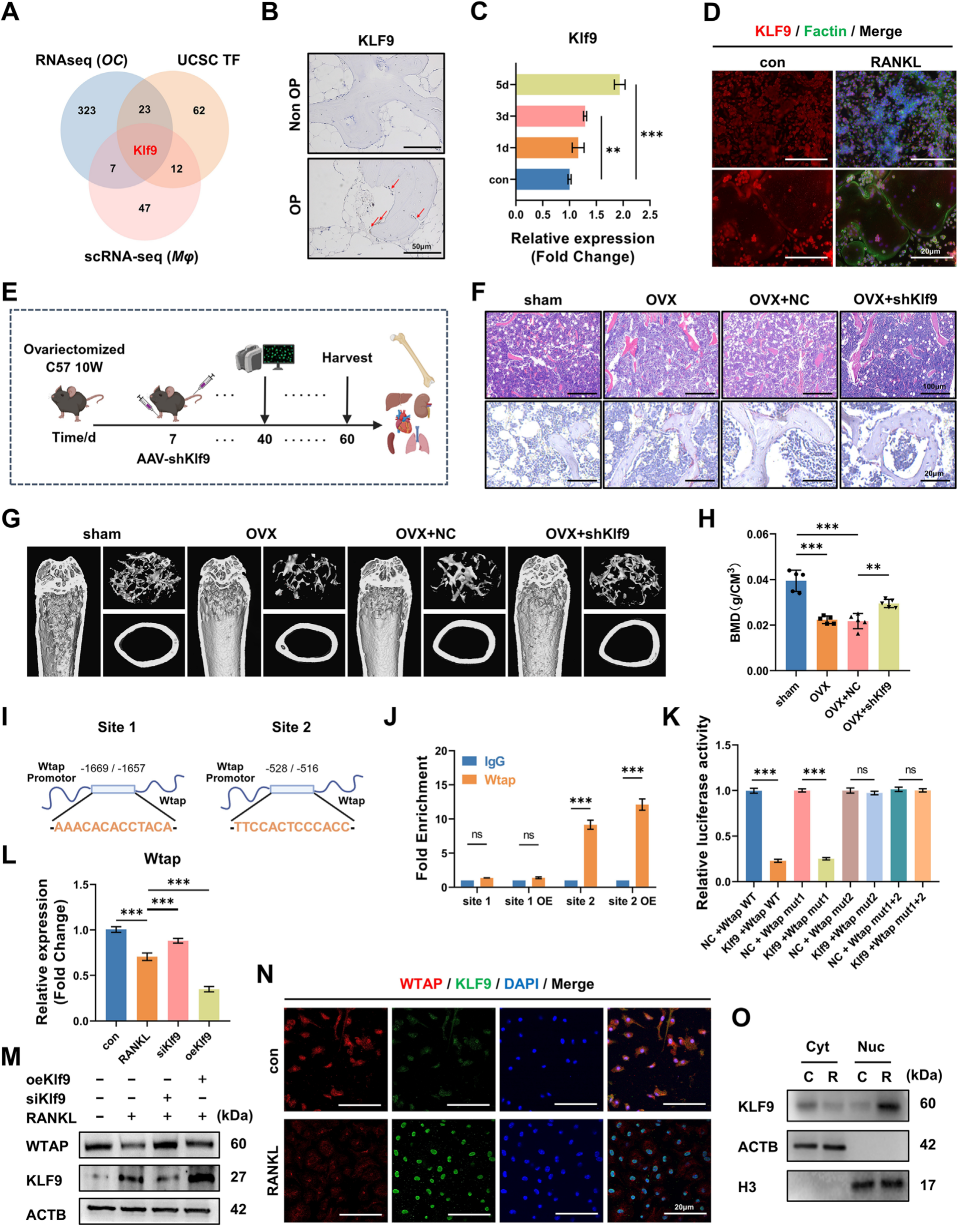
KLF9 acted as an upstream transcriptional repressor of WTAP to regulate osteoclastogenesis and OP progression via direct DNA binding after nuclear translocation. (A) Venn diagram of the intersection of DEGs from RNA‐seq of osteoclast differentiation (OC), transcription factor (TFs) binding sites predicted by the UCSC Genome Browser, and DEGs from scRNA‐seq of osteoclast precursors (Mφ). (B) Representative immunohistochemical staining images of KLF9 in bone tissue sections from non‐OP and OP patients. Scale bars, 50 µm. (C) qRT‐PCR analysis showing the relative expression of Klf9 mRNA in BMDMs at different time points after RANKL induction. (D) Representative immunofluorescence images showing the co‐localization of KLF9 (red) and F‐actin (green) in BMDMs treated with or without RANKL. Scale bars, 20 µm. (E) Schematic diagram of the experimental design for in vivo knockdown of Klf9 using AAV‐shKLF9 in OVX mice. (F) Representative H&E and TRAP staining images of bone tissue sections from sham, OVX, OVX + AAV‐NC, and OVX + AAV‐shKLF9 mice. Scale bars, 100 µm. (G) Representative micro‐CT images of the distal femur microstructure from the specific groups. (H) Quantification of bone mineral density (BMD) from micro‐CT analysis. (I) Schematic representation of the Wtap promoter region showing the predicted KLF9 binding motifs (Site 1: −1669 to −1657 bp; Site 2: −528 to −516 bp). (J) ChIP‐PCR showing the enrichment of KLF9 binding to the WTAP promoter at Site 1 and Site 2 in BMDMs overexpressing WTAP. (K) Dual‐luciferase reporter assay showing the relative luciferase activity of the Wtap promoter with wild‐type (WT) or mutated (mut1, mut2, mut1+2) KLF9 binding sites in the presence of Klf9 overexpression. (L) qRT‐PCR analysis of Wtap expression in BMDMs under KLF9 knockdown or overexpression. (M) Western blot analysis of WTAP and KLF9 protein levels in BMDMs under specific interventions. (N) Representative immunofluorescence images showing the subcellular localization of WTAP (red) and KLF9 (green) in BMDMs treated with or without RANKL. Scale bars, 20 µm. (O) Western blot analysis of KLF9 protein levels in cytoplasmic (Cyt) and nuclear (Nuc) fractions of BMDMs treated with or without RANKL. Data are presented as mean ± SEM. Statistical significance was determined by unpaired two‐tailed Student's *t*‐test (J) or one‐way ANOVA with Tukey's post hoc test (H, K, L). ^**^ *p* < 0.01, ^***^ *p* < 0.001, ns, not significant.

We first observed that KLF9 expression was elevated in bone tissue samples from OP patients compared to non‐OP controls (Figure 5B; Figure S10A). Consistent with the human data, KLF9 expression also increased in OVX mice. Immunofluorescence co‐localization studies revealed that KLF9 protein was expressed in cells positive for C‐fos (Figure S10B). In mice BMDMs undergoing RANKL‐induced differentiation, KLF9 mRNA levels were upregulated (Figure 5C). Western blot analysis further demonstrated that KLF9 protein expression increased during osteoclastogenesis, exhibiting a profile similar to, but initiating slightly earlier than, the induction of the osteoclast markers CTSK and MMP9 (Figure S10C–E). Immunofluorescence staining confirmed that KLF9 protein levels were substantially higher in mature, multinucleated osteoclasts (Figure 5D). Gain‐ and loss‐of‐function experiments using siRNA and overexpression plasmids established KLF9 as a positive regulator of osteoclast differentiation. KLF9 knockdown significantly inhibited the expression of osteoclastogenic markers and suppressed the formation and maturation of multinucleated osteoclasts (Figure S11A–C). Conversely, KLF9 overexpression robustly enhanced the expression of these markers and potently promoted osteoclastogenesis (Figure S11D–F). Pit formation assays further confirmed that KLF9 positively regulated osteoclast function. KLF9 knockdown substantially reduced the resorbed area, while KLF9 overexpression markedly increased it (Figure S11G). We next validated the function of KLF9 in human osteoclasts. Knockdown of KLF9 in hPBMCs significantly reduced the expression of osteoclastogenic markers and CSF1R at both mRNA and protein levels, while KLF9 overexpression exerted the opposite effect (Figure S12A,B). Meanwhile, WTAP expression showed the opposite upon Klf9 intervention. TRAP staining further demonstrated that Klf9 silencing impaired, and Klf9 overexpression promoted, human osteoclast formation (Figure S12C). These data confirmed that KLF9 positively regulated human osteoclast differentiation, mirroring the phenotype observed in mouse BMDMs.

To extend our findings to an in vivo context, AAV‐shKLF9 was administered via both tail vein and intraosseous injection, specifically targeting osteoclast precursor cells, after OVX surgery to generate a KLF9 knockdown mouse OP model (Figure 5E; Figure S13B). No visceral organ damage or abnormalities were observed during the animal experiment window (Figure S13A). The intervention efficiency of AAV was verified by immunofluorescence and Western blotting in animal tissues (Figure S13C,D). Compared to the AAV‐NC group, AAV‐shKLF9 group mice exhibited a significantly attenuated osteoporotic bone loss phenotype as assessed histologically (Figure 5F). Concomitantly, osteoclast activity was markedly suppressed, evidenced by reduced MMP9‐positive signals (Figure S13J). Mechanical testing demonstrated that AAV‐shKLF9 treatment significantly increased femoral ultimate load relative to AAV‐NC controls, indicating improved bone strength upon KLF9 knockdown (Figure S13E). Micro‐CT analysis revealed that AAV‐shKLF9 group mice displayed a significantly less severe OP phenotype compared to the AAV‐NC group (Figure 5G). This was characterized by higher bone mineral density (BMD), increased trabecular bone volume fraction (BV/TV), and greater trabecular number (Tb.N) (Figure 5H; Figure S13F,G). No significant differences were observed in trabecular separation (Tb.Sp) or cortical thickness (Ct.Th) between the two groups (Figure S13H,I). In summary, these data characterize KLF9, as a potential upstream transcriptional regulator of WTAP that can effectively modulate osteoclast differentiation and OP progression.

### 2.6 KLF9 Transcriptionally Represses WTAP Expression via Direct DNA Binding after Nuclear Translocation

Given that KLF9 functions as a transcription factor, we utilized the JASPAR database to predict potential KLF9 binding motifs upstream of the promoter region of the WTAP gene. Bioinformatic analysis using JASPAR identified two high‐confidence consensus KLF9 binding motifs upstream of the Wtap promoter region: one located at positions −1669 to −1657 bp (‐AAACACACCTACA‐) and the other at −528 to −516 bp (‐TTCCACTCCCACC‐) upstream of the transcription start site (Figure 5I). ChIP‐PCR using a KLF9 antibody confirmed the direct binding of KLF9 to the Wtap promoter. Enrichment was detected specifically at the predicted binding site 2 (−528 to −516 bp), but not at site 1 (−1669 to −1657 bp) (Figure 5J). Dual‐luciferase reporter assays demonstrated that KLF9 exerted a transcriptional repressive effect on the WTAP promoter. This repression was eliminated when the specific KLF9 binding site 2 was mutated, confirming that the inhibitory effect was mediated through direct binding at this motif (Figure 5K). To determine whether the weaker Site 1 (−1669 to −1657 bp) also contributed to KLF9‐mediated repression, we generated a double‐mutant reporter (mut1+2) in which both predicted binding sites were disrupted. Luciferase assays revealed that the double‐mutant construct behaved identically to the Site 2 single mutant: KLF9 overexpression failed to repress reporter activity in either case, and the mut1+2 construct showed no additional effect beyond the mutant on Site 2 alone. These results establish that Site 2 is the sole functional KLF9 binding site governing Wtap transcriptional repression. From a functional perspective, KLF9 knockdown attenuated the RANKL‐induced downregulation of Wtap expression, whereas KLF9 overexpression enhanced it. This reciprocal regulation of Wtap mRNA and protein levels by KLF9 was confirmed by qPCR and Western blot (Figure 5L,M). All these data suggested that KLF9 directly bound to the Wtap promoter at a specific motif and functioned as a transcriptional repressor to downregulate Wtap expression. We also observed that RANKL‐induced osteoclastogenesis of BMDMs not only increased KLF9 expression, but also triggered its nuclear translocation. Immunofluorescence microscopy and subcellular fractionation followed by Western blotting confirmed that KLF9, predominantly cytoplasmic in immature BMDMs, accumulated in the nucleus following RANKL induction (Figure 5N,O). These findings indicated that the transcription factor KLF9 played a critical role in the transcriptional repression of Wtap. Furthermore, they highlighted the potential of KLF9 as an important upstream transcriptional regulator within the gene network during osteoclast differentiation and maturation.

### 2.7 KLF9 Regulated Osteoclastogenesis and OP Progression via a WTAP‐Dependent Pathway

To determine whether KLF9 regulates osteoclastogenesis and OP by modulating WTAP, we performed rescue experiments both in vivo and in vitro. Specifically, we injected AAV‐shKLF9 into tamoxifen‐induced WTAP CKO mice and WTAP Ctrl mice to generate a double‐intervention model during OVX‐induced OP (Figure 6A). H&E staining confirmed that WTAP CKO mice injected with AAV‐NC (WTAP CKO + AAV‐NC) exhibited a more severe osteoporotic phenotype than the control group mice (WTAP Ctrl + AAV‐NC). Intervention with AAV‐shKLF9 significantly improved the bone phenotype in WTAP Ctrl mice (WTAP Ctrl + AAV‐shKLF9). In contrast, the bone loss in WTAP CKO mice was not rescued by AAV‐shKLF9 treatment (WTAP CKO + AAV‐shKLF9) (Figure 6B). Consistent with the histological findings, TRAP staining and immunohistochemistry for MMP9 revealed a parallel trend: osteoclast activity was elevated in WTAP CKO mice and suppressed by AAV‐shKLF9 in WTAP Ctrl mice, but AAV‐shKLF9 failed to inhibit osteoclast activity in the WTAP CKO background (Figure 6C,D). This functional outcome was further reflected at the systemic level, as serum CTX‐1 concentrations mirrored these findings (Figure 6E). Micro‐CT quantification provided definitive structural validation (Figure 6F). With the exception of cortical thickness (Ct.Th), trabecular bone parameters, including BMD, BV/TV, Tb.N, and Tb.Sp, were all improved by AAV‐shKLF9 treatment in WTAP Ctrl mice to some extent. Crucially, this beneficial effect of KLF9 knockdown on bone quality and bone microstructure was almost entirely abolished in WTAP CKO mice (Figure 6G; Figure S14A–E).

**FIGURE 6:**
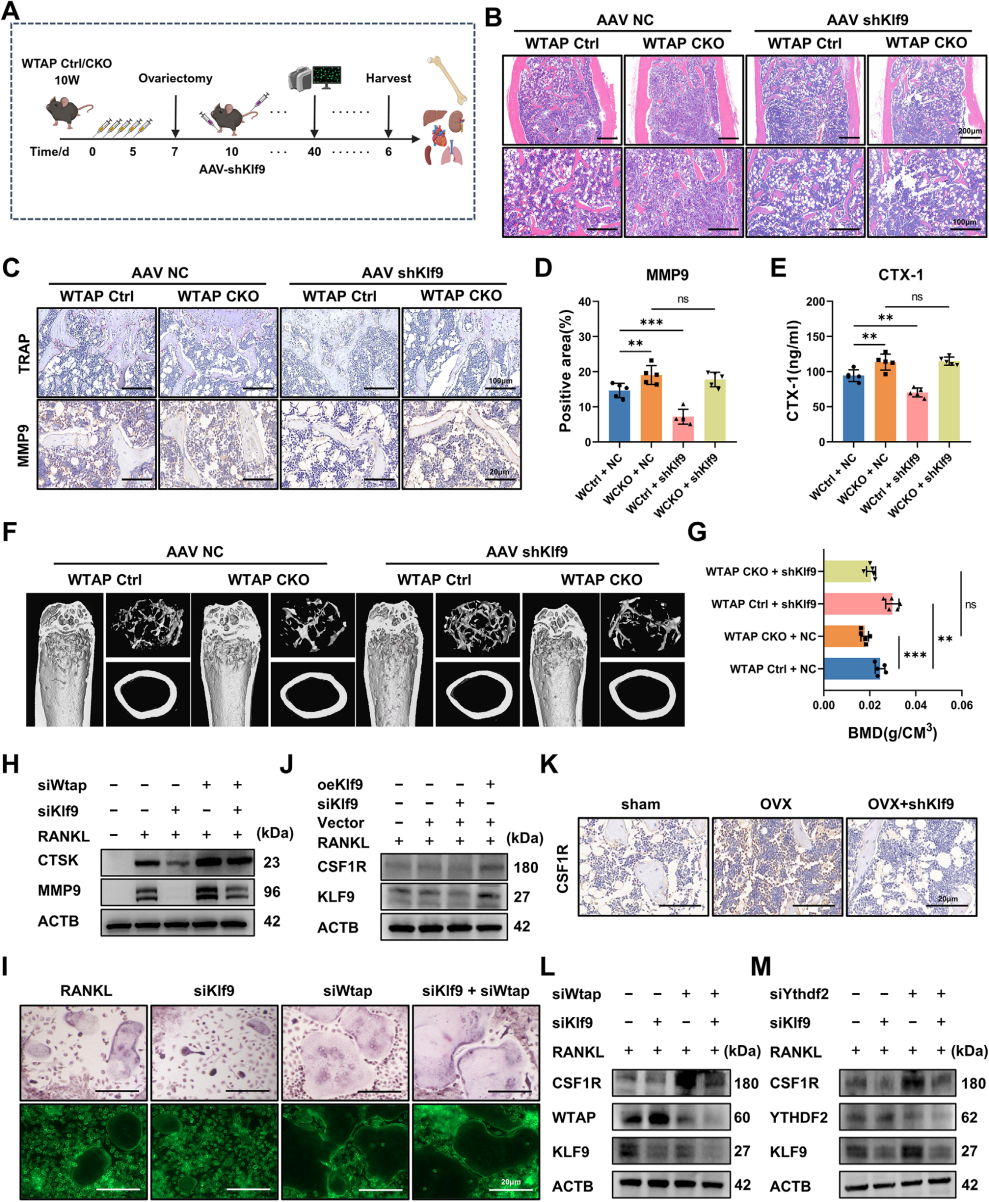
KLF9 regulated osteoclastogenesis and OP progression via a WTAP‐dependent pathway. (A) Schematic diagram of the experimental design for generating a double‐intervention mouse model. Tamoxifen‐induced WTAP CKO and WTAP Ctrl mice underwent OVX and were treated with both tail vein and intraosseous injections of AAV‐shKLF9 or AAV‐NC. (B) Representative H&E staining images of bone tissue sections from the indicated groups. Scale bars: 200 µm and 100 µm. (C) Representative TRAP and MMP9 staining images of bone tissue sections from the indicated groups. Scale bars, 100 µm. (D) Quantification of MMP9‐positive area in bone tissue sections (*n* = 5 per group). (E) Serum levels of CTX‐1 in the indicated groups (*n* = 5 per group). (F) Representative micro‐CT images of the distal femur from the indicated groups. (G) Quantification of bone mineral density (BMD) from micro‐CT analysis (*n* = 5 per group). (H) Western blot analysis of CTSK and MMP9 protein levels in BMDMs under siRNA interventions. (I) Representative TRAP staining and immunofluorescence images of osteoclasts formed under siRNA interventions. Scale bars, 20 µm. (J) Western blot analysis of CSF1R and KLF9 protein levels in BMDMs under overexpression or knockdown interventions. (K) Representative immunohistochemical staining images of CSF1R in bone tissue sections from sham, OVX, and OVX + AAV‐shKLF9 mice. Scale bars, 100 µm. L. Western blot analysis of CSF1R protein levels in BMDMs under siRNA interventions. (M) Western blot analysis of CSF1R, WTAP, and YTHDF2 protein levels in BMDMs under siRNA interventions. Data are presented as mean ± SEM. Statistical significance was determined by one‐way ANOVA with Tukey's post hoc test (D, E, G). ^**^ *p* < 0.01, ^***^ *p* < 0.001, ns, not significant.

Consistent with the in vivo data, the in vitro inhibitory effect of KLF9 knockdown on osteoclast differentiation (Ctsk, Mmp9) was abrogated in BMDMs where Wtap was concurrently knocked down (Figure 6H; Figure S15A). The same reverse effect was observed in functional assays of multinucleated osteoclast formation and maturation (Figure 6I; Figure S15B,C). Furthermore, manipulation of KLF9 expression (knockdown or overexpression) exerted a corresponding regulatory effect on CSF1R expression at both the mRNA and protein levels (Figure 6J; Figure S15D). In mice treated with AAV‐shKLF9, immunohistochemical staining revealed fewer CSF1R‐positive signals in bone tissue compared to the AAV‐NC control group (Figure 6K; Figure S15E). We also observed that the downregulation of CSF1R induced by KLF9 knockdown was abolished when WTAP was co‐depleted (Figure 6L, Figure S15F), which confirmed that KLF9 regulated CSF1R expression through a mechanism that was functionally dependent on WTAP. Notably, similar effects were observed in hPBMCs upon co‐knockdown of KLF9 and WTAP (Figure S16A–C), indicating that KLF9 functioned upstream of WTAP in the human osteoclast lineage. Crucially, knockdown of Ythdf2 similarly abrogated the suppression of CSF1R expression caused by KLF9 knockdown (Figure 6M; Figure S15G). This demonstrated that the effect of KLF9 on CSF1R is executed through the WTAP‐YTHDF2 regulatory pathway.

Collectively, these data prompted a regulatory pathway in which KLF9 promoted osteoclast differentiation by transcriptionally repressing WTAP, thereby attenuating the WTAP/YTHDF2/m^6^A‐mediated decay of CSF1R mRNA and elevating CSF1R protein expression.

### 2.8 Targeting the WTAP‐CSF1R Axis in Osteoclast Precursors Ameliorated OVX‐Induced Osteoporosis

To evaluate the therapeutic potential of modulating the WTAP‐CSF1R axis for OP, we performed t interventional experiments in wild‐type mice. We constructed an AAV designed to overexpress WTAP specifically in osteoclast precursors and administered it to mice in an OVX‐induced OP model (Figure 7A; Figure S17B). No visceral organ damage or abnormalities were observed (Figure S17A). The intervention efficiency of AAV was verified by immunofluorescence and Western blotting in bone tissues (Figure S17C,D). H&E staining revealed that treatment with AAV‐oeWtap partially alleviated the osteoporotic bone loss phenotype in OVX mice compared to AAV‐NC mice (Figure 7B). Consistent with the improved bone quality, both TRAP staining and MMP9 immunohistochemistry demonstrated that AAV‐oeWTAP treatment reduced osteoclast number and resorptive activity on bone surfaces (Figure 7C,D). This reduction in bone resorption was further corroborated by a decrease in serum levels of CTX‐1 (Figure 7E). Three‐point bending analysis showed that AAV‐oeWTAP treatment partially restored femoral maximum load in OVX mice compared with the AAV‐NC group (Figure S17E). Micro‐CT confirmed the therapeutic effect, showing significant improvements in key trabecular bone parameters after AAV‐oeWTAP intervention (Figure 7F–G; Figure S17F–I).

**FIGURE 7:**
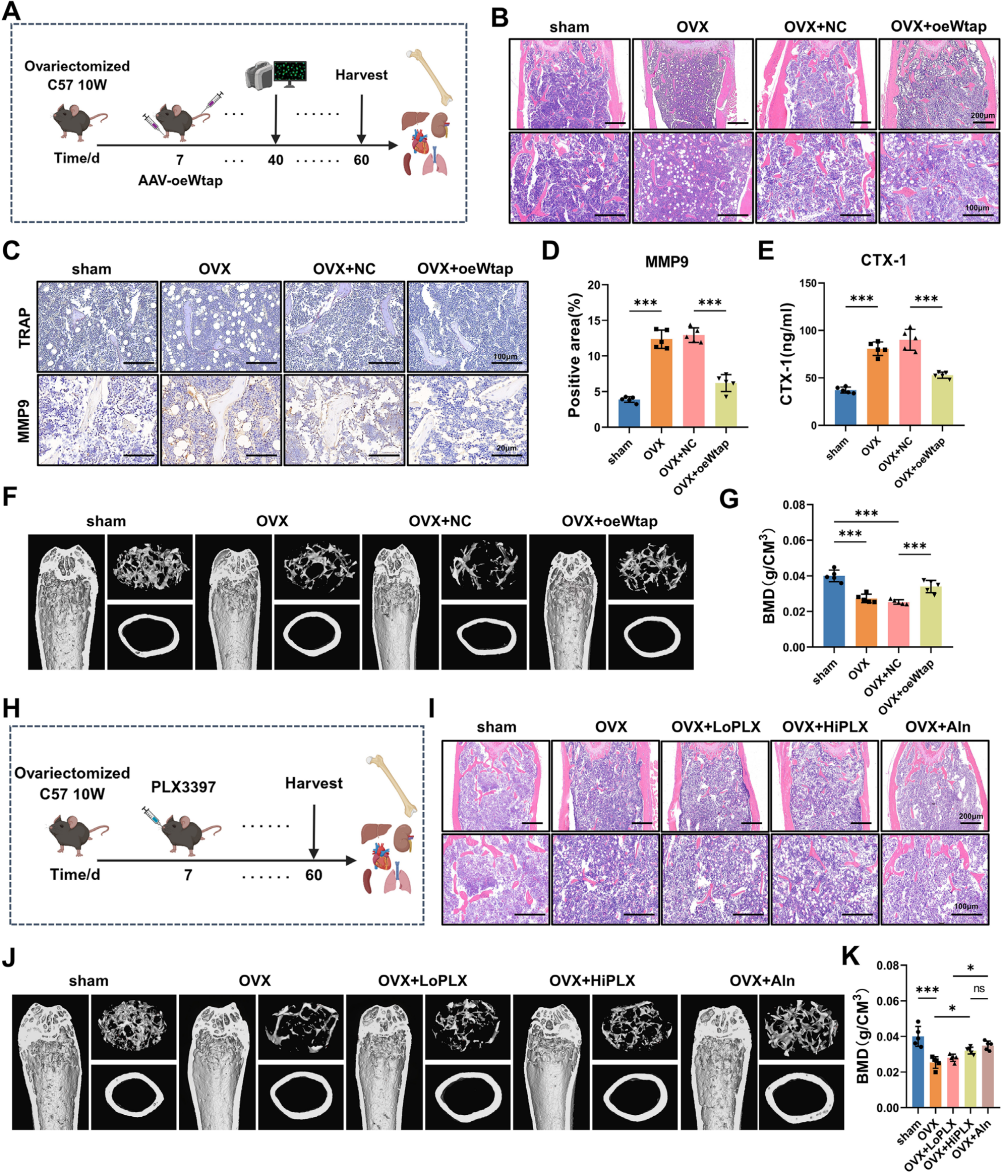
Targeting the WTAP‐CSF1R axis in osteoclast precursors ameliorated OVX‐induced osteoporosis. (A) Schematic diagram of the experimental design for the AAV‐mediated intervention. OVX C57 mice were injected with AAV‐oeWtap or AAV‐NC on day 7 post‐surgery and harvested on day 60. B. Representative H&E staining images of bone tissue sections from the indicated groups. Scale bars: 200 µm and 100 µm. (C) Representative TRAP and MMP9 staining images of bone tissue sections from the indicated groups. Scale bars, 100 µm. (D) Quantification of MMP9‐positive area in bone tissue sections (*n* = 5 per group). E. Serum levels of CTX‐1 in the indicated groups (*n* = 5 per group). (F) Representative micro‐CT images of the distal femur from the indicated groups. (G) Quantification of bone mineral density (BMD) from micro‐CT analysis (*n* = 5 per group). (H) Schematic diagram of the experimental design for the pharmacological intervention with PLX3397. OVX C57 mice were administered PLX3397 or vehicle on day 7 post‐surgery and harvested on day 60. (I) Representative H&E staining images of bone tissue sections from the indicated groups. Scale bars: 200 µm and 100 µm. (J) Representative micro‐CT images of the distal femur from the indicated groups. (K) Quantification of bone mineral density (BMD) from micro‐CT analysis (*n* = 5 per group). Data are presented as mean ± SEM. Statistical significance was determined by one‐way ANOVA with Tukey's post hoc test (D, E, G, K). ^*^ *p* < 0.05, ^***^ *p* < 0.001.

To pharmacologically target the WTAP‐CSF1R axis, we identified the FDA‐approved small‐molecule inhibitor pexidartinib (PLX3397), a potent and selective antagonist of CSF1R (Figure 7H). Previous studies have demonstrated that PLX3397 potently inhibited osteoclast differentiation in vitro [28]. It has been shown to effectively mitigate LPS‐induced OP and improve outcomes of delayed fracture healing by suppressing osteoclast‐mediated bone resorption [29, 30]. As a positive control, an additional cohort received alendronate (ALN, 0.1 mg/kg every 2 days, oral gavage). In our study, we found that PLX3397 administration ameliorated the osteoporotic bone loss phenotype in OVX mice in a dose‐dependent manner (Figure 7I) without visceral organ damage or abnormalities observed (Figure S18A). PLX3397 administration improved femoral maximum load in a dose‐dependent manner, with the high‐dose group showing a significant increase compared with vehicle‐treated OVX controls (Figure S18B). Micro‐CT analysis confirmed the dose‐dependent improvement in trabecular bone parameters following PLX3397 treatment (Figures 7J‐7K, Figure S18C–F). The high‐dose PLX3397 group approached the efficacy of ALN, although the ALN group maintained a modestly superior effect on certain microstructural parameters. Notably, while both therapies ameliorated osteoporotic bone loss, CSF1R inhibition interrupts osteoclastogenesis at the signaling level, thereby avoiding the rebound in bone turnover that is often observed following bisphosphonate discontinuation, which is a distinguishing feature with implications for long‐term management. These data highlighted the therapeutic potential of CSF1R inhibitor PLX3397 for treating postmenopausal OP.

## 3 Discussion

Excessive bone resorption driven by osteoclasts leads to the pathological progression in OP and related metabolic bone diseases [31]. Although the RANKL‐RANK and M‐CSF‐CSF1R signaling pathways are recognized as master regulators of osteoclast differentiation, the upstream networks controlling these pathways, particularly at the post‐transcriptional level, remain incompletely understood. Here, we discover and systematically characterize a previously unknown regulatory axis, KLF9/WTAP/YTHDF2/m^6^A/CSF1R, that plays a vital role in osteoclastogenesis and bone homeostasis in OP (Figure 8). We demonstrate that the transcription factor KLF9, upregulated during osteoclastogenesis, acts as a direct transcriptional repressor of m^6^A “Writer” WTAP, whose downregulation reduces m^6^A deposition on CSF1R mRNA. This decrease in m^6^A modification attenuates the degradation of CSF1R transcripts by the m^6^A “Reader” YTHDF2, resulting in the stabilization of CSF1R mRNA and consequent elevation of CSF1R protein levels. This molecular mechanism ultimately enhances M‐CSF‐CSF1R signaling, driving excessive osteoclast formation and bone loss in estrogen‐deficient OP. Our findings not only reveal a novel epitranscriptomic mechanism controlling the expression of a classic ligand‐receptor signal, but also demonstrate the distinctive cellular function of WTAP in bone metabolism. More importantly, these mechanistic insights show a translational therapeutic potential: intervention via WTAP gene delivery in osteoclast precursors or pharmacological inhibition of the downstream target CSF1R could effectively mitigate osteoporotic bone loss in preclinical models, thereby paving the way for novel targeted strategies to treat pathological bone resorption.

**FIGURE 8:**
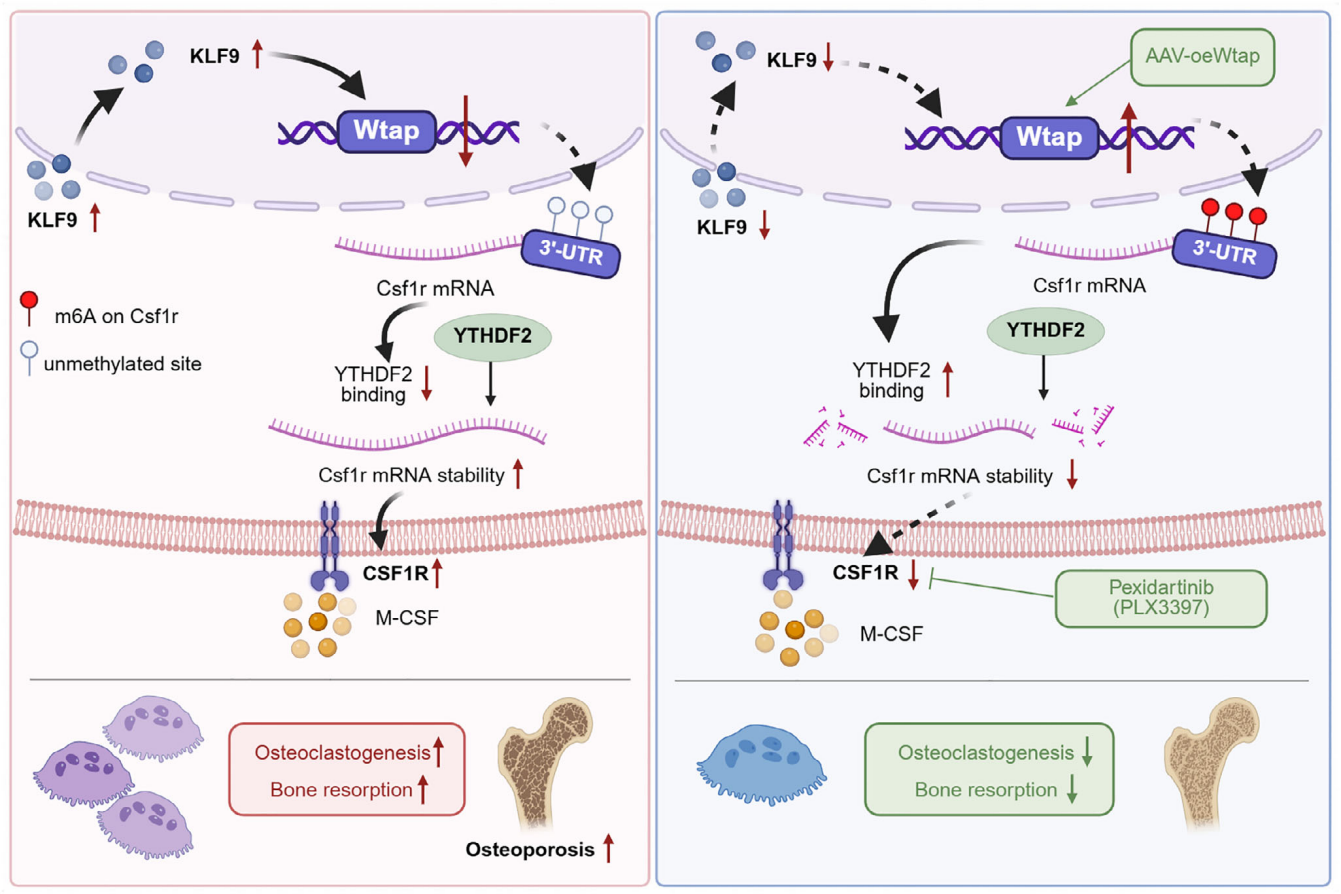
Scheme of the KLF9/WTAP/YTHDF2/m^6^A/CSF1R regulatory axis in osteoclastogenesis and estrogen‐deficient osteoporosis. WTAP‐mediated m^6^A modification of CSF1R mRNA governs osteoclastogenesis via a YTHDF2‐mediated mechanism. Pathological upregulation of KLF9 drives Wtap transcription, leading to increased m^6^A deposition on the 3’‐UTR of Csf1r mRNA. This reduces YTHDF2 binding affinity, destabilizing CSF1R mRNA and elevating CSF1R protein levels upon M‐CSF stimulation, thereby enhancing osteoclastogenesis and promoting bone resorption. Conversely, therapeutic inhibition of KLF9 or WTAP decreases m6A modification on CSF1R, restores YTHDF2 binding, stabilizes CSF1R mRNA, and reduces CSF1R expression, ultimately suppressing osteoclast differentiation. Pharmacological blockade of CSF1R with PLX3397 further attenuates osteoclastogenesis, providing a strategy to alleviate estrogen‐deficient osteoporosis.

The m^6^A modification regulates a sophisticated regulatory network in osteoclast‐mediated bone resorption in bone homeostasis, involving the synergistic action of “Writer”, “Eraser” and “Reader” proteins. Our study finds that “Writer” WTAP absence stabilizes CSF1R expression and promotes osteoclast formation by reducing m^6^A modification of CSF1R mRNA and weakening YTHDF2‐mediated degradation, consistent with a previous study that WTAP knockdown leads to increased osteoclast differentiation. However, METTL3 and METTL14, which are both m^6^A methyltransferase complex subunits, exhibit different regulatory modes. METTL3 has been reported to enhance osteoclast differentiation by stabilizing CHI3L1 mRNA [32] or by increasing the sponging effect of circ_0008542on miR‐185‐5p [33], thereby derepressing RANK signaling. In contrast, METTL14 overexpression inhibits osteoclast formation by promoting YTHDF2‐mediated decay of Nfatc1 mRNA [14]. Other “Writer” proteins, like KIAA1429, have also been found to suppress osteoclastogenesis via the m^6^A/YTHDC1/LRP4 axis [34]. In addition, m^6^A regulatory proteins, including the “Erasers” FTO, as well as “Readers” from the YTHDC and YTHDF families, have also been implicated in diverse and context‐dependent roles in osteoclast biology [35, 36, 37]. These findings align with our observation that RANKL stimulation does not induce a global shift in m^6^A levels; instead, it elicits gene‐specific increases and decreases in methylation. This pattern underscores the fine‐tuned, target‐specific division of modular nature of the m^6^A regulatory network during osteoclast differentiation.

One noteworthy aspect is the central role of WTAP, which lacks intrinsic methyltransferase activity. As a core regulatory subunit of the m^6^A methyltransferase complex, WTAP functions as an essential scaffold and localization factor [38]. Its primary roles include stabilizing the catalytic METTL3‐METTL14 heterodimer, facilitating the recruitment of the entire complex to specific nuclear speckles, and guiding the complex to its target mRNAs [39]. Evidence indicates that the RNA‐binding capacity of METTL3 is severely compromised in the absence of WTAP, suggesting WTAP's indispensable role in substrate recognition and complex integrity [40]. Furthermore, WTAP can bridge other RNA‐binding proteins, such as RBM15, to the catalytic core, thereby integrating additional specificity into the m^6^A deposition process. Therefore, WTAP operates as a master conductor of the m^6^A “Writer” complex, revealing its assembly, subcellular localization, and target selection, which ultimately dictates the functional outcome, such as the suppression of Csf1r mRNA stability and osteoclastogenesis, as demonstrated in our study. Nevertheless, further experimental studies are required to determine whether WTAP exerts functions independent of the METTL3‐METTL14 catalytic core and whether it plays additional, novel roles in cellular physiology.

Interestingly, despite the marked changes in “Writers”, the global m^6^A modification levels in BMDMs remained relatively stable throughout RANKL‐induced osteoclast differentiation. This phenomenon is consistent with the notion that RANKL, as a cytokine ligand, does not directly modify m6A machinery; rather, it orchestrates a balanced, transcript‐specific rewiring of m6A marks. During osteoclastogenesis, the concurrent downregulation of Wtap and dynamic shifts in Mettl3/Mettl14 expression led to coordinated gains and losses of m6A peaks across the transcriptome, resulting in a net equilibrium at the global level. In contrast, forced Wtap overexpression introduces a supraphysiological abundance of the m^6^A “Writer” complex, breaking this homeostatic balance and thereby elevating global m^6^A levels. This dynamic equilibrium in m^6^A methylation is reminiscent of the target‐specific regulation observed for other m^6^A writers [41].

While the downstream effects of m^6^A modification in post‐transcriptional gene regulation during osteoclastogenesis are well‐established, the upstream transcriptional control of the m^6^A mechanism itself remains unclearly explored. Our study identifies KLF9 as a direct transcriptional repressor of Wtap, which provides another critical regulatory approach that considers transcription factors as master regulators of the osteoclast epitranscriptome. This emphasizes that m^6^A methylation is not a passive executor but is dynamically regulated at the transcriptional level, integrating upstream signals to modulate osteoclast fate. Emerging evidence proves that the expression of m^6^A “Writers” is subject to tight transcriptional control. For instance, transcription factor EGR1 promotes the transcription of Mettl3 to drive osteoclast differentiation [32]. In hepatocellular carcinoma, STAT3 has been shown to directly bind to the Wtap promoter and upregulate its expression [42]. Besides transcription factors, epigenetic modifications at the histone and DNA levels also participate in the regulation of m^6^A‐related genes, showing the potential to modulate osteoclastogenesis [43]. METTL3 acetylation regulates its nuclear/cytosolic functions to restrain cancer metastasis [44]. Histone lactylation of m^6^A regulatory genes also influences their activity, stability, and localization, thereby playing important roles in diverse processes including tumor progression and therapy resistance [45, 46, 47, 48]. DNA methylation is also involved. In pancreatic ductal adenocarcinoma cells, cigarette smoke condensate promotes the transcription of the Mettl3 gene by reducing the methylation level of the CpG islands [49]. These findings collectively underscore the regulatory potential upstream of m^6^A‐associated genes, positioning their transcriptional control as an important aspect in regulating the m^6^A epitranscriptome [50].

These mechanisms suggest the existence of a multi‐level epigenetic‐epitranscriptomic crosstalk. A prime example is Nfatc1, a master regulator of osteoclastogenesis. METTL14‐mediated m^6^A methylation at a specific site (4249A) on Nfatc1 mRNA promotes its degradation via YTHDF2, thereby inhibiting osteoclast differentiation [14]. This creates a precise regulatory circuit: upstream signals (e.g., RANKL) induce transcription factors that regulate m^6^A “Writers”, which in turn post‐transcriptionally tune the levels of critical transcription factors like Nfatc1, ultimately controlling the osteoclast differentiation outcome. This provides new insights for targeted m^6^A modification therapy. Targeting upstream transcriptional regulators or epigenetic modifiers could offer a strategic approach to indirectly normalize dysregulated m^6^A‐dependent pathways in OP, potentially with greater precision than broadly targeting the core m^6^A complex.

Our study has several limitations. First, although our aged mouse model did not reveal a statistically significant deterioration of the osteoporotic phenotype in WTAP CKO mice, a trend toward worsening was observed. This may be related to the specific induction protocol or observation window applied. Further optimization of the aging model is needed, and the precise role of WTAP in senile osteoporosis remains an important aspect for future investigation. Second, KLF9 is identified as a transcriptional repressor of Wtap after nuclear translocation; the precise upstream signals regulating KLF9 expression and activation remain unclear. Elucidating these is essential to understand the mechanism's initiation. The inherent limitations and specificity of the Lyz2‐CreERT2 system used to generate myeloid‐specific Wtap knockout mice remained a problem, which needs further exploration in genetically engineered mouse system construction. Finally, while the CSF1R inhibitor PLX3397 showed efficacy, its systemic use may affect tissue‐resident macrophages. Developing bone‐targeted delivery systems or osteoclast‐precursor‐specific nanocarriers is crucial to enhance specificity before clinical translation [51]. It should also be noted that the human bone tissue data presented in this study are correlative in nature. The observed inverse relationship between WTAP expression and serum CTX‐1 levels indicates an association between reduced WTAP abundance and increased bone resorption, but does not establish causality. The causal role of WTAP in regulating osteoclastogenesis and bone loss is further needed via a randomized clinical trial under ethical approval in the future.

## 4 Conclusion

To sum up, our study elucidates a novel KLF9/WTAP/YTHDF2/m^6^A/CSF1R regulatory axis governing osteoclastogenesis and OP progression. We demonstrate that the transcription factor KLF9, induced during osteoclast differentiation, transcriptionally represses WTAP. Reduced WTAP expression decreases m^6^A methylation on CSF1R mRNA, thereby attenuating its YTHDF2‐mediated decay and elevating CSF1R protein levels, which ultimately regulates osteoclast formation. These findings uncover a critical epitranscriptomic mechanism controlling osteoclast‐mediated bone resorption and highlight the therapeutic potential of targeting this pathway, as evidenced by the efficacy of the CSF1R inhibitor PLX3397.

## 5 Methods

### 5.1 Human Sample Collection

Bone tissue samples were collected from 16 female patients (8 with OP and 8 non‐OP controls) undergoing total knee arthroplasty at The First Affiliated Hospital of Soochow University, with informed consent and approval from the Institutional Review Board (No. 2026312). The diagnosis of OP was based on dual‐energy X‐ray absorptiometry (DEXA) with a bone mineral density (BMD) T‐score≤‐2.5 at the neck of the femur. The average age of patients with OP is 67.75 ± 2.82, while the average age of non‐OP patients is 66.50 ± 4.38. No statistically significant difference was found between the two groups. The average BMD T‐score of OP patients is −2.78 ± 0.35, while the non‐OP group is −0.93 ± 0.64. Following the surgery, the patient's tissue samples were fixed in 4% paraformaldehyde for subsequent histological analysis. Preoperative blood samples were collected for the assessment of serum C‐terminal telopeptide of type I (CTX‐1) levels, which were measured using an ELISA kit (D711143, Sangon Biotech, Shanghai, China).

### 5.2 Animal Models

All animal experiments were approved by the Institutional Animal Care and Use Committee (IACUC) of Soochow University (No. SUDA20260507A04). C57BL/6J mice (8–10 weeks old, female) were purchased from Beijing Vital River Laboratory Animal Technology Co., Ltd. The ovariectomy (OVX) induced OP model was established by bilateral ovariectomy under anesthesia. Sham‐operated mice underwent the same procedure without ovary removal. Wtap^flox/flox^ mice (S‐CKO‐12549) and Lyz2‐CreERT2 mice (C001499) were purchased from Cyagen Biosciences Inc. The myeloid‐specific WTAP conditional knockout (WTAP CKO) mice were generated by crossing WTAP^FLOX/flox^ mice with Lyz2‐CreERT2 mice. Age‐matched WTAP^FLOX/+^; Lyz2‐CreERT2 littermates served as controls (WTAP Ctrl). Mouse genotypes were determined by PCR followed by agarose gel electrophoresis (Table S1). Gene knockout was induced in 8‐week‐old mice by intraperitoneal injection of tamoxifen (75 mg/kg/day for 5 consecutive days). Mice were sacrificed 8 weeks after OVX surgery, whose femurs and major visceral organs (lung, liver, spleen, kidney, heart) were harvested for histological evaluation (*n* = 5 per group). Micro‐CT was applied to validate the effectiveness of the model. For the senile OP model, mice were aged to 10 months under standard housing conditions. Gene knockout was induced by intraperitoneal injection of tamoxifen (75 mg/kg/day for 5 consecutive days). Mice were sacrificed 6 months after tamoxifen induction, and their femurs and main visceral organs were harvested for histological evaluation (*n* = 5 per group).

### 5.3 Adeno‐Associated Virus (AAV) and Drug Administration

AAV‐shKlf9 and AAV‐oeWtap were conducted by Shanghai Obio Technology (Group) Corp., Ltd. For KLF9 knockdown in vivo, AAV‐shKLF9 or a non‐targeting control (AAV‐NC) was administered via tail vein injection and intraosseous injection (5 × 10^1^ ^1^ viral genomes per mouse) 7 days after OVX surgery. The same approach was applied to Wtap overexpression using AAV‐oeWtap. Intervention efficiency was confirmed by immunofluorescence and Western blotting of BMDMs. Mice were sacrificed 8 weeks after OVX surgery, and their femurs and major visceral organs (lung, liver, spleen, kidney, heart) were harvested for histological evaluation.

For pharmacological intervention, the CSF1R inhibitor pexidartinib (PLX3397) (HY‐16749, MedChemExpress, United States) was dissolved in corn oil and administered via oral gavage once every 2 days starting from 7 days after OVX surgery. The low‐ and high‐dose groups received drug administrations at concentrations of 10 mg/kg (LoPLX) and 30 mg/kg (HiPLX), respectively. Control mice received vehicle (corn oil) only. As a positive control, OVX mice received alendronate sodium (ALN, Sigma‐Aldrich, United States) at 0.1 mg/kg body weight via oral gavage every 2 days for 8 weeks. Mice were sacrificed 8 weeks after OVX surgery, and their femurs and main visceral organs (lung, liver, spleen, kidney, heart) were harvested for histological evaluation.

### 5.4 Three‐Point Bending Test

Femoral bone strength was assessed by three‐point bending using a mechanical testing machine (Instron 5944, USA). Femora were dissected free of soft tissue and stored in PBS‐soaked gauze at −20°C until testing. On the day of testing, bones were thawed to room temperature and positioned on two supports spaced 8 mm apart with the anterior surface facing upward. A preload of 1 N was applied, followed by a compressive load at a constant speed of 0.5 mm/min until fracture. The ultimate load (N) at failure was recorded (*n* = 8 per group). Discard the femur sample after testing.

### 5.5 Micro‐CT Analysis

The distal femurs were scanned using a high‐resolution micro‐CT system (SkyScan, Bruker, Belgium) with the following parameters: source voltage 50 kV, current 500 µA, pixel size 9 µm. 3D reconstruction and analysis of trabecular bone parameters, including bone mineral density (BMD), bone volume fraction (BV/TV), trabecular number (Tb.N), trabecular separation (Tb.Sp), and cortical thickness (Ct.Th), were performed using CTAn software.

### 5.6 Histology and Immunohistochemistry

Bone tissues were fixed in 4% paraformaldehyde and embedded in paraffin. Sections (5 µm) were stained with Hematoxylin and Eosin (H&E) (G1120, Solarbio, Beijing, China) for general histology or with tartrate‐resistant acid phosphatase (TRAP) (G1492, Solarbio, Beijing, China) for osteoclast detection. For immunohistochemistry, sections were subjected to antigen retrieval, blocked, and incubated with primary antibodies against WTAP (A22751, Abclonal, Wuhan, China), MMP9 (10375‐2‐AP, Proteintech, Wuhan, China), KLF9 (A7196, Abclonal, Wuhan, China), or CSF1R (A3019, Abclonal, Wuhan, China) overnight at 4°C. Signals were developed using a DAB substrate kit and counterstained with hematoxylin (36312ES, Yeasen Biotechnology, Shanghai, China). Images were captured using a microscope (Zeiss, Germany) and analyzed with ImageJ.

### 5.7 Cell Culture and Osteoclastogenesis Induction

Primary bone marrow‐derived macrophages (BMDMs) were isolated from the femurs of 6‐week‐old mice by flushing the bone marrow cavity with α‐MEM (Cytiva, United States) with 1% penicillin‐streptomycin (RG‐CE‐19, Ketu Biotech, Hefei, China). Cells were cultured in α‐MEM supplemented with 10% fetal bovine serum (FBS) (CG‐SR‐02, Ketu Biotech, Hefei, China), and 30 ng/mL recombinant macrophage colony‐stimulating factor (M‐CSF) (R&D Systems, United States) for 3 days. For osteoclast differentiation, BMDMs were seeded and stimulated with 50 ng/mL M‐CSF and 50 ng/mL recombinant RANKL (R&D Systems, United States) for 5–7 days. The medium was replaced every 2 days. DPBS (RG‐CE‐15, Ketu Biotech, China) was used to rinse the cells. After cell harvest, the total m^6^A modification level was detected by ELISA (EpiGentek, United States). The separation of nuclei and cytoplasmic proteins was performed (P0027, Beyotime, Shanghai, China).

### 5.8 Human Peripheral Blood Monocyte Isolation and Osteoclast Differentiation

Peripheral blood was collected from healthy volunteers, approved by the Institutional Review Board of the First Affiliated Hospital of Soochow University (No. 2026312). The isolation procedure was referred to the current protocol [52]. Isolated cells were seeded and cultured in α‐MEM supplemented with 10% FBS and 50 ng/mL M‐CSF for 1 day. Osteoclast differentiation was induced by adding 50 ng/mL M‐CSF and 50 ng/mL RANKL (R&D Systems, United States) for 10 days, with medium replacement every 3 days.

### 5.9 Bone Resorption Assay

Osteoclast‐mediated bone resorption was evaluated using bone slices. Mature osteoclasts were generated from BMDMs as described above and reseeded onto cell culture plates. After 14d of co‐culture, cells were removed by incubation with 10% sodium hypochlorite for 10 min. Resorption pits were imaged under bright‐field microscopy, and the resorbed area was quantified. Data are presented as a percentage of total surface area resorbed.

### 5.10 Osteoblast Differentiation of BMSCs

Primary bone marrow mesenchymal stem cells (BMSCs) were isolated from femurs of 6‐week‐old C57BL/6 mice and cultured in α‐MEM containing 10% FBS and 1% penicillin/streptomycin. For osteogenic induction, BMSCs were seeded and cultured in osteogenic medium (α‐MEM supplemented with 10% FBS, 50 µg/mL ascorbic acid, 10 mm β‐glycerophosphate, and 100 nm dexamethasone). The medium was changed every 2 days. ALP staining was performed on day 10 using an ALP staining kit (C3206, Beyotime, Shanghai, China) according to the manufacturer's instructions. Alizarin Red S staining was performed on day 21; cells were fixed with 4% paraformaldehyde, stained with 2% Alizarin Red S solution (pH = 4.2) (C0138, Beyotime, Shanghai, China), and washed with PBS. Mineralized nodules were visualized and photographed under a light microscope.

### 5.11 Gene Knockdown and Overexpression

Small interfering RNA (siRNA) targeting Wtap, Klf9, Ythdf2, or non‐targeting control siRNA (Sangon Biotech, Shanghai, China) was transfected into BMDMs using HighGene Transfection Reagent (RM09014, Abclonal, Wuhan, China) according to the manufacturer's protocol. The siRNA sequences are shown in Table S2. For overexpression, the full‐length coding sequences of Wtap or KLF9 were cloned into the pcDNA3.1 vector (Sangon Biotech, Shanghai, China). Plasmids were transfected into BMDMs using HighGene Transfection Reagent. Cells were harvested 48–72 h post‐transfection for subsequent experiments. Knockdown and overexpression efficiencies were validated by quantitative real‐time PCR (qRT‐PCR) and Western blotting.

### 5.12 Methylated RNA Immunoprecipitation Sequencing (MeRIP‐seq) and RNA Sequencing (RNA‐seq)

MeRIP‐seq and RNA‐seq were performed on BMDMs overexpressing WTAP by Lc‐Bio Technologies (Hangzhou) Co., Ltd. RNA‐seq was performed on BMDMs derived from WTAP CKO and WTAP Ctrl mice by HaploX Biotechnology Co., Ltd. Total RNA from AAV‐oeWTAP/AAV‐NC treated BMDMs and WTAP CKO/WTAP Ctrl BMDMs was isolated, quality‐checked, and used to prepare strand‐specific libraries via poly(A) enrichment, fragmentation, and adapter ligation. Libraries were sequenced (PE150) on an Illumina NovaSeq 6000. Raw reads were trimmed, aligned to the mouse genome, and analyzed for differential expression (*p* < 0.05, |log2FC| ≥ 1) using DESeq2.

For AAV‐oeWTAP BMDMs, total RNA was fragmented. M^6^A‐modified RNA was immunoprecipitated with an anti‐m^6^A antibody, competitively eluted, and used (with input RNA) to construct sequencing libraries. IP and input libraries were sequenced. Peaks were called, and differential methylation (*p* < 0.05, |log2FC| ≥ 1) was analyzed. Data were integrated with RNA‐seq to identify genes with coordinated changes in expression and m^6^A modification.

### 5.13 Bioinformatic Analysis

Single‐cell RNA sequencing (scRNA‐seq) data from human osteoporotic bone samples (GSE147287) and bulk RNA‐seq data from RANKL‐induced osteoclast differentiation (GSE246769) were downloaded from the Gene Expression Omnibus (GEO) database. Data processing, normalization, and differential expression analysis were performed using the Seurat and DESeq2 R packages, respectively. Pseudotime trajectory analysis was conducted with Monocle3. For m^6^A methylomic analysis, m^6^A peak calling and differential methylation analysis were performed using exomePeak2. Gene Ontology (GO) enrichment analysis was performed using the clusterProfiler R package. Potential transcription factor binding sites were predicted using the UCSC genome browser and JASPAR database.

### 5.14 Immunofluorescence

Cells grown on coverslips or frozen bone sections were fixed, rehydrated, and blocked. Samples were incubated with primary antibodies against WTAP (A22751, Abclonal, Wuhan, China, 60188‐1‐lg, Proteintech, Wuhan, China), KLF9 (A7196, Abclonal, Wuhan, China), YTHDF2(A25318, Abclonal, Wuhan, China), C‐fos (66590‐1‐lg, Proteintech, Wuhan, China) overnight at 4°C, followed by appropriate Alexa Fluor‐conjugated secondary antibodies (Beyotime, Shanghai, China). Nuclei were counterstained with DAPI (Beyotime, Shanghai, China). Images were acquired using a microscope (Zeiss, Germany) and a confocal laser scanning microscope (Leica, Germany) and analyzed using ImageJ.

### 5.15 Quantitative PCR and Western Blotting

Total RNA was extracted from cells or tissues using Freezol reagent (Vazyme, Nanjing, China). Complementary DNA (cDNA) was synthesized using the PrimeScript RT reagent Kit (Vazyme, Nanjing, China). qRT‐PCR was performed using SYBR Green Master Mix (Vazyme, Nanjing, China). Gene expression levels were normalized to Actb and calculated using the 2^−ΔΔCt^ method. Primer sequences are listed in Table S3.

Proteins were extracted from BMDMs using RIPA lysis buffer (Beyotime, Shanghai, China) containing protease and phosphatase inhibitors. Equal amounts of protein were separated by SDS‐PAGE and transferred onto NC membranes. After blocking, membranes were incubated overnight at 4°C with primary antibodies against ACTB (AC026, Abclonal, Wuhan, China), CTSK (A1782, Abclonal, Wuhan, China), MMP9, WTAP, KLF9, YTHDF2, and CSF1R. After incubation with HRP‐conjugated secondary antibodies (Beyotime, Shanghai, China), protein bands were visualized using an enhanced chemiluminescence (ECL) detection system. Quantitative analysis was performed using ImageJ.

### 5.16 Methylated RNA Immunoprecipitation‐qPCR (MeRIP‐PCR)

Total RNA was extracted and fragmented using RNA fragmentation buffer. Fragmented RNA was incubated with an anti‐WTAP antibody or normal rabbit IgG (control) conjugated to protein A/G magnetic beads overnight at 4°C. After washing, bound RNA was eluted and purified (Bes5203, Bersinbio Bioscience, Guangzhou, China). The enrichment of m^6^A‐modified Csf1r mRNA was quantified by qPCR. Input RNA (1%) was used for normalization.

### 5.17 RNA Stability Assay

BMDMs were treated with 5 µg/mL Actinomycin D (HY‐17559, MedChemExpress, United States) to block transcription. Cells were harvested at indicated time points (0 h, 2 h, 4 h, 6 h, 8 h). Total RNA was extracted, and the remaining Csf1r mRNA levels were quantified by qPCR.

### 5.18 RNA Immunoprecipitation‐qPCR (RIP‐PCR)

Total RNA was extracted and fragmented using RNA fragmentation buffer. Fragmented RNA was incubated with an anti‐YTHDF2 antibody or normal rabbit IgG (control) conjugated to protein magnetic beads overnight at 4°C. After washing, bound RNA was extracted and reverse transcribed (Bes5101, Bersinbio Bioscience, Guangzhou, China). The enrichment of Csf1r mRNA was analyzed by qPCR. Input RNA (1%) was used for normalization.

### 5.19 Chromatin Immunoprecipitation‐qPCR (ChIP‐qPCR)

BMDMs were harvested and immobilized on Concanavalin A‐coated magnetic beads. After permeabilization, cells were incubated with an anti‐KLF9 antibody or normal rabbit IgG (control) overnight at 4°C, followed by incubation with a secondary antibody and the pA/G‐Tn5 transposome complex. Tagmentation was performed at 37°C for 1 h to cleave and tag the chromatin. DNA was then extracted using the kit's purification system. The purified DNA was used as a template for quantitative PCR (qPCR) with primers specific to the Wtap promoter region (Table S4). The enrichment of KLF9 binding was calculated relative to the input DNA and normalized to the IgG control group (TD904, Vazyme Technology, Nanjing, China).

### 5.20 Dual‐Luciferase Reporter Assay

The promoter region of the Wtap gene (wild‐type or with mutations in the KLF9 binding site) was cloned into the pGL3‐Basic luciferase reporter vector. BMDMs were co‐transfected with the reporter plasmid, a Renilla luciferase control plasmid (pRL‐TK), and either a KLF9 overexpression plasmid or an empty vector (Sangon Biotech, Shanghai, China). Luciferase activity was measured 48 h post‐transfection using the Dual‐Luciferase Reporter Assay System (RG009, Beyotime, Shanghai, China). Firefly luciferase activity was normalized to Renilla luciferase activity.

### 5.21 Statistical Analysis

All data are presented as mean ± standard deviation (SD). Statistical analyses were performed using GraphPad Prism 8.0. For comparisons between two groups, an unpaired two‐tailed Student's *t*‐test was used. For comparisons among multiple groups, one‐way or two‐way analysis of variance (ANOVA) followed by Tukey's post‐hoc test was used. Correlation analysis was performed using Pearson's correlation coefficient. A *p*‐value of less than 0.05 was considered statistically significant.

## Author Contributions

Conceptualization: CS, HLY, DCG; Methodology: CS, XL; Formal analysis: CS, LX; Validation: CS, XL, LX, ZYZ; Investigation: CL, HX, WHL, YQ; Data Curation: QFS, QHW; Supervision: GRG, JZ; Funding acquisition: GRG, HXL, HLY, DCG; Project administration: HLY, DCG; Writing – original draft: CS, GRG; Writing – review & editing: CS, HLY, DCG.

## Funding

This work was supported by the National Natural Science Foundation of China (82472525, 82272567, 82470272 and 82502941), Jiangsu Medical Research Project (ZD2022014), Program of Suzhou Health Commission (GSWS2022002), National and Local Engineering Laboratory of New Functional Polymer Materials (SDGC2205), Foundation of National Center for Translational Medicine (Shanghai) SHU Branch (SUITM‐202403), the Project of MOE Key Laboratory of Geriatric Diseases and Immunology (No. KJS2502), the Priority Academic Program Development of Jiangsu Higher Education Institutions (PAPD), Suzhou Basic Research Youth Program (SSD2025027), Science and Education Project of Suzhou (QNXM2024001) and BoXi Talent Cultivation Program (BXQN2024006).

## Conflicts of Interest

The authors declare no conflicts of interest.

## Supporting information




**Supporting File**: advs77227‐sup‐0001‐SuppMat.docx.

## Data Availability

The data that support the findings of this study are available on request from the corresponding author. The data are not publicly available due to privacy or ethical restrictions.
