# Supplementary material for: WTAP Transcriptional Suppression by KLF9 Drives Osteoclastogenesis via M6A‐Mediated Regulation of CSF1R Signaling in Estrogen‐Deficient Osteoporosis
Source: Adv Sci (Weinh). 2026 Aug 30;13(60):e77227. doi: 10.1002/advs.77227 (PMC13526421; doi:10.1002/advs.77227)
Supplement: Supplementary file 1 [file ADVS-13-e77227-s001.docx]

**Supporting Information**

**Title: WTAP Transcriptional Suppression by KLF9 Drives Osteoclastogenesis via m^6^A-Mediated Regulation of CSF1R Signaling in Estrogen-Deficient Osteoporosis**

*Chen Shen, Xin Liu, Gaoran Ge, Li Xu, Ziyu Zhang, Chang Lv, Hao Xu, Wenhao Li, Yi Qin, Qifeng Sheng, Qihan Wang, Hongxia Li^*^, Jun Zhou^*^, Huilin Yang^*^, Dechun Geng^*^*

**Table S1: Primer sequences for mouse genotyping.**

| Primer | Sequences (5’-3’) |
| --- | --- |
| WTAP^flox/flox^ F1 | AACAGGGTATATTCAAGTTGGCCTC |
| WTAP^flox/flox^ R1 | AAAGACCCAGGTAGTGGTAATGTT |
| Lyz2-CreERT2 P1 | TGAGGAAAAACAGAAGCCATTATT |
| Lyz2-CreERT2 P3 | CTTTCGCTTTCCCCCTCCCTATTG |
| Lyz2-CreERT2 P4 | ACTGCTCCCCTGTCCCTGTTCTGA |

**Table S2: Sequences of siRNA.**

| siRNA | Sequences (5’-3’) |
| --- | --- |
| Wtap-1-s | CGGGAUGAGUUAAUUCUAA/dT//dT/ |
| Wtap-1-a | UUAGAAUUAACUCAUCCCG/dT//dT/ |
| Wtap-2-s | AAACGAUGUGACUGGUUUA/dT//dT/ |
| Wtap-2-a | UAAACCAGUCACAUCGUUU/dT//dT/ |
| Wtap-3-s | GCAAGAGUGCACCACUCAA/dT//dT/ |
| Wtap-3-a | UUGAGUGGUGCACUCUUGC/dT//dT/ |
| Klf9-1-s | AGAGCUUGUUGGACCUCAA/dT//dT/ |
| Klf9-1-a | UUGAGGUCCAACAAGCUCU/dT//dT/ |
| Klf9-2-s | GGACCUCAACAAAUACCGA/dT//dT/ |
| Klf9-2-a | UCGGUAUUUGUUGAGGUCC/dT//dT/ |
| Klf9-3-s | CAGCAUGAUCAAGAGAUCA/dT//dT/ |
| Klf9-3-a | UGAUCUCUUGAUCAUGCUG/dT//dT/ |
| Ythdf2-1-s | GGAUGGACUAAAUGAUGAU/dT//dT/ |
| Ythdf2-1-a | AUCAUCAUUUAGUCCAUCC/dT//dT/ |
| Ythdf2-2-s | CCUACUUACCCAGUUACUA/dT//dT/ |
| Ythdf2-2-a | UAGUAACUGGGUAAGUAGG/dT//dT/ |
| Ythdf2-3-s | GGUGAAGCUGCUUGGUCUA/dT//dT/ |
| Ythdf2-3-a | UAGACCAAGCAGCUUCACC/dT//dT/ |
| Csf1r-s | GGAAGAUCAUCGAGAGAUA/dT//dT/ |
| Csf1r-a | UAUCUCUCGAUGAUCUUCC/dT//dT/ |
| Wtap-s (homo) | GGUUCGAUUGAGUGAAACA/dT//dT/ |
| Wtap-a (homo) | UGUUUCACUCAAUCGAACC/dT//dT/ |
| Klf9-s (homo) | CGACAGUCUGGAAAGUCCA/dT//dT/ |
| Klf9-a (homo) | UGGACUUUCCAGACUGUCG/dT//dT/ |

**Table S3: Primer sequences for qRT-PCR**

| Primer | Sequences (5’-3’) |
| --- | --- |
| Actb-F | CTACCTCATGAAGATCCTGACC |
| Actb-R | CACAGCTTCTCTTTGATGTCAC |
| Acp5-F | CAAGAACTTGCGACCATTGTTA |
| Acp5-R | ATCCATAGTGAAACCGCAAGTA |
| Nfatc1-F | GAGAATCGAGATCACCTCCTAC |
| Nfatc1-R | TTGCAGCTAGGAAGTACGTCTT |
| Ctsk-F | GCTTGGCATCTTTCCAGTTTTA |
| Ctsk-R | CAACACTGCATGGTTCACATTA |
| Mmp9-F | CAAAGACCTGAAAACCTCCAAC |
| Mmp9-R | GACTGCTTCTCTCCCATCATC |
| Wtap-F | TGAGATCAACAATGGTAGACCC |
| Wtap-R | TTGGTTCTCCTGGATAAGCATT |
| Mettl3-F | CGCTGCCTCCGATGTTGATCTG |
| Mettl3-R | CCTGACTGACCTTCTTGCTCTGC |
| Mettl14-F | ACATCTGCTCCAAACTCAAAAC |
| Mettl14-R | CAGTGTCTACAAAATGTTGGCA |
| Klf9-F | ATTATTGCACGCTGGTCACTAT |
| Klf9-R | ATCCTATATCCTCATCGGGACT |
| Csf1r-F | CGAAGTGGGATTCAACGTTATC |
| Csf1r-R | CCACACAAGAATATATGCCAGC |
| Ythdf2-F | CCCCCAATAAAGCATAACATGG |
| Ythdf2-R | TGGGTTGGCTGACCTATATTTT |
| Runx2-F | CCTTCAAGGTTGTAGCCCTC |
| Runx2-R | GGAGTAGTTCTCATCATTCCCG |
| Bglap-F | GGACCATCTTTCTGCTCAC |
| Bglap-R | ACATGAAGGCTTTGTCAGAC |
|  |  |
| Col1a1-F | TGAACGTGGTGTACAAGGTC |
| Col1a1-R | CCATCTTTACCAGGAGAACCAT |
| Nfatc1-F (homo) | CACCGCATCACAGGGAAGAC |
| Nfatc1-R (homo) | GCACAGTCAATGACGGCTC |
| Ctsk-F (homo) | ACACCCACTGGGAGCTATG |
| Ctsk-R (homo) | GACAGGGGTACTTTGAGTCCA |
| Mmp9-F (homo) | TGTACCGCTATGGTTACACTCG |
| Mmp9-R (homo) | GGCAGGGACAGTTGCTTCT |
| Csf1r-F (homo) | GGGAATCCCAGTGATAGAGCC |
| Csf1r-R (homo) | TTGGAAGGTAGCGTTGTTGGT |

**Table S4: Primer sequences for Wtap promotor binding region**

| Primer (mus) | Sequences (5’-3’) |
| --- | --- |
| 1669.1-F | ACAGAAGTACAGTGGGATGCAA |
| 1669.1-R | TGCATACTAAAGTCCCGGAGC |
| 1669.2-F | CAGTGGGATGCAAAACACAC |
| 1669.2-R | TTGGATTTAGGACCTTCTGCGA |
| 528.1-F | TGTACTCCGTGGAACTTGAACC |
| 528.1-R | GAAAGTCGCTCCACCTCACTT |
| 528.2-F | TACTCCGTGGAACTTGAACCAT |
| 528.2-R | TAGAAAGTCGCTCCACCTCAC |

**Figure S1:** A. tSNE plots showing the expression of Mettl3 and Mettl14 in single-cell RNA sequencing data from OP patient bone marrow. B. Pseudotemporal trajectory analysis revealing the relative expression of Mettl3 and Mettl14 during osteoclast differentiation. C. Pseudotemporal trajectory analysis showing the relative expression of Wtap during BMSC differentiation. D. Quantification of WTAP positive area in bone tissue sections from sham-operated (Ctrl) and OVX mice (n = 5 per group). E. Serum CTX-1 levels in Ctrl and OVX mice (n = 5 per group). F. Correlation analysis between WTAP expression in bone tissue and serum CTX-1 levels in mice. Data are presented as mean ± SEM. Statistical significance was determined by unpaired two-tailed Student’s t-test (D, E). ****p* < 0.001.

**Figure S2:** A. qRT-PCR analysis of osteoclast marker genes (Acp5, Nfatc1, Ctsk, Mmp9) in BMDMs at different time points after RANKL stimulation. B. qRT-PCR analysis of Mettl3 and Mettl14 expression in BMDMs at different time points after RANKL stimulation. C. Quantification of WTAP protein levels in BMDMs at different time points after RANKL stimulation. D. Quantification of CTSK and MMP9 protein levels in BMDMs at different time points after RANKL stimulation. E. Quantification of relative m^6^A modification in total RNA from BMDMs treated with or without RANKL. F. Quantification of relative m^6^A modification in 200 ng total RNA from BMDMs treated with or without RANKL. Data are presented as mean ± SEM. Statistical significance was determined by unpaired two-tailed Student’s t-test (E, F) or one-way ANOVA with Tukey’s post hoc test (A, B, C, D). **p* < 0.05, ***p* < 0.01, ****p* < 0.001, ns, not significant.

**Figure S3:** A. Quantification of WTAP protein levels in BMDMs transfected with NC or Wtap siRNAs. B. Quantification of CTSK and MMP9 protein levels in BMDMs after Wtap knockdown. C. Quantification of multinucleated cell (MNC) rate in BMDMs under siWtap intervention. D. Quantification of cell fusion area in BMDMs after Wtap knockdown. E. Quantification of WTAP protein levels in BMDMs transfected with vector control or Wtap overexpression plasmid. F. Quantification of CTSK and MMP9 protein levels in BMDMs after Wtap overexpression. G. Quantification of MNC rate in BMDMs under oeWtap intervention. H. Quantification of cell fusion area in BMDMs after Wtap overexpression. I. Representative bright-field images of resorption pits formed by BMDMs transfected with vector control, siWtap, or Wtap overexpression plasmid and quantification of the resorbed pit area as a percentage of total surface area (n = 3 per group). Scale bar, 100 μm. Data are presented as mean ± SEM. Statistical significance was determined by unpaired two-tailed Student’s t-test (C, D, G, H) or one-way ANOVA with Tukey’s post hoc test (A, B, E, F, I). **p* < 0.05, ***p* < 0.01, ****p* < 0.001, ns, not significant.

**Figure S4:** A. Representative images of ALP staining and Alizarin Red S staining in BMSCs after Wtap knockdown. B. qRT-PCR analysis of osteoblast marker genes (Runx2, Bglap, Col1a1) in BMSCs under siWtap intervention. Data are presented as mean ± SEM. Statistical significance was determined by one-way ANOVA with Tukey’s post hoc test (B). **p* < 0.05, ***p* < 0.01, ****p* < 0.001.

**Figure S5:** A. Schematic diagram of the generation of WTAP CKO mice by crossing Wtap^flox/flox^ mice with Lyz2-CreERT2 mice. B. Genotyping PCR results confirming the presence of the Wtap^flox/flox^ allele and the Lyz2-CreERT2 transgene in representative mice. C. Western blot analysis confirming the efficient knockout of WTAP protein in BMDMs derived from WTAP CKO mice compared to WTAP Ctrl mice. D. Representative immunofluorescence images showing the specific deletion of WTAP (green) in bone marrow cells of WTAP CKO mice. Scale bars, 50 μm. E. Representative H&E staining images of major visceral organs (lung, liver, spleen, kidney, heart) from WTAP Ctrl and WTAP CKO mice, showing no apparent pathological abnormalities. Scale bars, 100 μm. F. Representative TRAP and MMP9 staining images of bone tissue sections from WTAP Ctrl and WTAP CKO mice. Scale bars, 50 μm. G. Quantification of MMP9 positive area in bone tissue sections (n = 5 per group). H. Representative TRAP staining images of BMDMs isolated from WTAP Ctrl and WTAP CKO mice after RANKL stimulation. Scale bars, 20 μm. I. Representative bright-field images of resorption pits formed by BMDMs from WTAP Ctrl and WTAP CKO mice and quantification of the resorbed pit area as a percentage of total surface area. Scale bar, 100 μm. J. Serum CTX-1 levels in WTAP Ctrl and WTAP CKO mice treated with sham or OVX surgery (n = 5 per group). K. Quantification of femoral ultimate load by three-point bending test in WTAP Ctrl and WTAP CKO mice under OVX conditions (n = 8 per group). L-O. Micro-CT quantification of trabecular bone parameters in the distal femur, including bone volume fraction (BV/TV), trabecular number (Tb.N), trabecular separation (Tb.Sp), and cortical thickness (Ct.Th) (n = 5 per group). Data are presented as mean ± SEM. Statistical significance was determined by unpaired two-tailed Student’s t-test (G) or one-way ANOVA with Tukey’s post hoc test (J, K, L, M, N, O). **p* < 0.05, ***p* < 0.01, ****p* < 0.001, ns, not significant.

**Figure S6:** A. Schematic diagram of the experimental timeline for assessing age-related bone loss in 10-month-old WTAP Ctrl and WTAP CKO mice, including tamoxifen induction and harvest at 16 months of age. B. Representative H&E staining images of femur sections from male and female WTAP Ctrl and WTAP CKO mice. Scale bars, 100 μm. C. Representative micro-CT images of the distal femur from male and female WTAP Ctrl and WTAP CKO mice. D. Serum CTX-1 levels in male and female WTAP Ctrl and WTAP CKO mice (n = 5 per group). E-I. Micro-CT quantification of bone parameters in the distal femur of male and female WTAP Ctrl and WTAP CKO mice, including bone mineral density (BMD), bone volume fraction (BV/TV), trabecular number (Tb.N), trabecular separation (Tb.Sp), and cortical thickness (Ct.Th) (n = 5 per group). Data are presented as mean ± SEM. Statistical significance was determined by unpaired two-tailed Student’s t-test (D, E, F, G, H, I). Ns, not significant.

**Figure S7:** A. Heatmap showing the top 100 DEGs and their corresponding m⁶A modification status in BMDMs overexpressing Wtap (OE) compared to control (Ran). The red box highlights Csf1r. B. Schematic diagram of the experimental design for RNA-seq analysis of BMDMs isolated from WTAP Ctrl and WTAP CKO mice. C. Volcano plot showing the distribution of DEGs in BMDMs from WTAP CKO mice compared to WTAP Ctrl mice. The red arrow indicates Csf1r. D. Heatmap showing the top 50 DEGs in BMDMs from WTAP CKO mice compared to WTAP Ctrl mice. The red box highlights Csf1r. E. Gene Ontology (GO) enrichment analysis of downregulated (left) and upregulated (right) genes in BMDMs from WTAP CKO mice.

**Figure S8:** A. qRT-PCR analysis showing the relative expression of Nfatc1, Ctsk, and Mmp9 in BMDMs transfected with vector, siWtap, or siWtap combined with siCsf1r. B. Western blot analysis of CSF1R, CTSK, and MMP9 protein levels after the indicated conditions. C. Representative TRAP staining images and quantification of MNC rate in BMDMs of osteoclasts formed under the indicated conditions. Scale bar, 20 μm. Data are presented as mean ± SEM. Statistical significance was determined by one-way ANOVA with Tukey’s post hoc test (A, C). **p* < 0.05, ***p* < 0.01, ****p* < 0.001.

**Figure S9:** A. qRT-PCR analysis of Nfatc1, Ctsk, Mmp9, and Csf1r mRNA levels by hPBMCs transfected with vector control, siWtap, or Wtap overexpression plasmid. B. Western blot analysis of CSF1R, CTSK, and MMP9 protein levels after the indicated conditions. C. Representative TRAP staining images in hPBMCs of osteoclasts formed under the indicated conditions. Scale bar, 50 μm. Data are presented as mean ± SEM. Statistical significance was determined by one-way ANOVA with Tukey’s post hoc test (A). **p* < 0.05, ***p* < 0.01, ****p* < 0.001.

**Figure S10:** A. Quantification of KLF9 positive cells in bone tissue sections from non-OP and OP patients (n = 5 per group). B. Representative immunofluorescence images showing the co-localization of KLF9 (green) and C-fos (red) in bone tissue sections from Ctrl and OVX mice. Scale bars, 50 μm. C. Western blot analysis showing the protein levels of KLF9, CTSK, and MMP9 in BMDMs at different time points during RANKL induction. D. Quantification of KLF9 protein level in BMDMs in C. E. Quantification of CTSK and MMP9 protein levels in C. Data are presented as mean ± SEM. Statistical significance was determined by unpaired two-tailed Student’s t-test (A) or one-way ANOVA with Tukey’s post hoc test (D). ****p* < 0.001.

**Figure S11:** A. qRT-PCR analysis showing the relative expression of Klf9, Nfatc1, Ctsk, and Mmp9 in BMDMs transfected with NC or Klf9 siRNA. B. Western blot analysis of KLF9, CTSK, and MMP9 protein levels in BMDMs after siKlf9 interventions. C. Representative TRAP staining and immunofluorescence images of osteoclasts formed under siKlf9 interventions. Scale bars, 20 μm. D. qRT-PCR analysis showing the relative expression of Klf9, Nfatc1, Ctsk, and Mmp9 in BMDMs treated transfected with vector control or Klf9 overexpression plasmid. E. Western blot analysis of KLF9, CTSK, and MMP9 protein levels in BMDMs after oeKlf9 interventions. F. Representative TRAP staining and immunofluorescence images of osteoclasts formed under oeKlf9 interventions. Scale bars, 20 μm. G. Representative bright-field images of resorption pits formed by BMDMs transfected with vector control, siWtap, or Wtap overexpression plasmid and quantification of the resorbed pit area as a percentage of total surface area (n = 3 per group). Scale bar, 100 μm. Data are presented as mean ± SEM. Statistical significance was determined by unpaired one-way ANOVA with Tukey’s post hoc test (A, D, G). **p* < 0.05, ***p* < 0.01, ****p* < 0.001, ns, not significant.

**Figure S12:** A. qRT-PCR analysis of Nfatc1, Ctsk, Mmp9, and Csf1r mRNA levels by hPBMCs transfected with vector control, siKlf9, or Klf9 overexpression plasmid. B. Western blot analysis of WTAP, CSF1R, CTSK, and MMP9 protein levels after the indicated conditions. C. Representative TRAP staining images in hPBMCs of osteoclasts formed under the indicated conditions. Scale bar, 50 μm. Data are presented as mean ± SEM. Statistical significance was determined by one-way ANOVA with Tukey’s post hoc test (A). **p* < 0.05, ***p* < 0.01.

**Figure S13:** A. Representative H&E staining images of major visceral organs (lung, liver, spleen, kidney, heart) from sham, OVX, OVX + AAV-NC, and OVX + AAV-shKlf9 mice. Scale bars, 100 μm. B. Schematic diagram of the AAV-Lyz2-ZsGreen-miRshKlf9 vector construct used for in vivo knockdown. C. Representative immunofluorescence images showing GFP expression in bone marrow cells from mice injected with AAV-NC or AAV-shKlf9. Scale bars, 50 μm. D. Western blot analysis validating the knockdown efficiency of KLF9 in BMDMs derived from AAV-shKlf9 mice compared to AAV-NC mice. E. Quantification of femoral ultimate load in sham, OVX, OVX + AAV-NC, and OVX + AAV-shKLF9 mice (n = 8 per group). F-I. Micro-CT quantification of trabecular bone parameters in the distal femur, including bone volume fraction (BV/TV), trabecular number (Tb.N), trabecular separation (Tb.Sp), and cortical thickness (Ct.Th) (n = 5 per group). J. Representative immunohistochemical staining images of MMP9 in bone tissue sections from the indicated groups. Scale bars, 100 μm. Data are presented as mean ± SEM. Statistical significance was determined by unpaired one-way ANOVA with Tukey’s post hoc test (E, F, G, H, I, J). **p* < 0.05, ***p* < 0.01, ****p* < 0.001, ns, not significant.

**Figure S14:** A. Quantification of femoral maximum load in the indicated groups (n = 8 per group). B-E. Micro-CT quantification of trabecular bone parameters in the distal femur, including bone volume fraction (BV/TV), trabecular number (Tb.N), trabecular separation (Tb.Sp), and cortical thickness (Ct.Th) (n = 5 per group). Data are presented as mean ± SEM. Statistical significance was determined by one-way ANOVA with Tukey’s post hoc test (A, B, C, D, E). **p* < 0.05, ***p* < 0.01, ns, not significant.

**Figure S15:** A. qRT-PCR analysis showing the relative expression of Ctsk and Mmp9 in BMDMs transfected with siRNAs. B. Quantification of the MNC rate in BMDMs under siRNA interventions. C. Quantification of the cell fusion area in BMDMs under siRNA interventions. D. qRT-PCR analysis showing the relative expression of Csf1r in BMDMs upon Klf9 knockdown or overexpression. E. Quantification of CSF1R positive area in bone tissue sections from sham, OVX, and OVX + AAV-shKlf9 mice (n = 5 per group). F. qRT-PCR analysis showing the relative expression of Csf1r in BMDMs under siRNAs interventions. G. qRT-PCR analysis showing the relative expression of Csf1r in BMDMs under siRNAs interventions. Data are presented as mean ± SEM. Statistical significance was determined by one-way ANOVA with Tukey’s post hoc test (A, B, C, D, E, F, G). **p* < 0.05, ***p* < 0.01, ****p* < 0.001.

**Figure S16:** A. qRT-PCR analysis of Nfatc1, Ctsk, Mmp9, and Csf1r mRNA levels by hPBMCs transfected with transfected with vector, siWtap, or siWtap combined with siKlf9. B. Western blot analysis of CSF1R, CTSK, and MMP9 protein levels after the indicated conditions. C. Representative TRAP staining images in hPBMCs of osteoclasts formed under the indicated conditions. Scale bar, 50 μm. Data are presented as mean ± SEM. Statistical significance was determined by one-way ANOVA with Tukey’s post hoc test (A). **p* < 0.05, ***p* < 0.01, *** *p* < 0.001.

**Figure S17:** A. Representative H&E staining images of major visceral organs (lung, liver, spleen, kidney, heart) from sham, OVX, OVX + NC, and OVX + oeWtap mice. Scale bars, 100 μm. B. Schematic diagram of the AAV-Lyz2-ZsGreen-Wtap vector construct used for in vivo overexpression. C. Representative immunofluorescence images showing GFP expression in bone marrow cells from mice injected with AAV-NC or AAV-oeWtap. Scale bars, 50 μm. D. Western blot analysis validating the overexpression efficiency of WTAP in BMDMs derived from AAV-oeWtap mice compared to AAV-NC mice. E. Quantification of femoral ultimate load in sham, OVX + AAV-NC, and OVX + AAV-oeWtap mice (n = 8 per group). F-I. Micro-CT quantification of trabecular bone parameters in the distal femur, including bone volume fraction (BV/TV), trabecular number (Tb.N), trabecular separation (Tb.Sp), and cortical thickness (Ct.Th) (n = 5 per group). Data are presented as mean ± SEM. Statistical significance was determined by one-way ANOVA with Tukey’s post hoc test (E, F, G, H, I). **p* < 0.05, ****p* < 0.001, ns, not significant.

**Figure S18:** A. Representative H&E staining images of major visceral organs (lung, liver, spleen, kidney, heart) from sham, OVX, OVX + LoPLX, OVX + HiPLX, and OVX + Aln mice. Scale bars, 100 μm. B. Quantification of femoral maximum load in sham, OVX + vehicle, OVX + LoPLX, OVX + HiPLX and OVX + Aln mice (n = 8 per group). C-F. Micro-CT quantification of trabecular bone parameters in the distal femur, including bone volume fraction (BV/TV), trabecular number (Tb.N), trabecular separation (Tb.Sp), and cortical thickness (Ct.Th) (n = 5 per group). Data are presented as mean ± SEM. Statistical significance was determined by one-way ANOVA with Tukey’s post hoc test (B, C, D, E, F). **p* < 0.05, ***p* < 0.01, ****p* < 0.001, ns, not significant.
